# A robust asymmetric bioadhesive patch for sutureless intestinal repair to prevent anastomotic leakage and postoperative adhesion

**DOI:** 10.7150/thno.135611

**Published:** 2026-06-17

**Authors:** Ruiqi Gao, Fangfang Su, Xiaohua Li, Zhongjie He, Yanglin Pan, Yaping Zheng, Mingzhong Wang, Lijun Yuan, Jiahe Liang, Xiaoqian Li

**Affiliations:** 1 Department of Ultrasound Medicine, Tangdu Hospital, The Fourth Military Medical University, Xi’an, Shaanxi, 710038, P. R. China.; 2 Department of Gastrointestinal Surgery, Xijing Hospital, The Fourth Military Medical University, Xi’an 710032, Shaanxi, P. R. China.; 3 Xijing Hospital of Digestive Diseases, The Fourth Military Medical University, Xi’an, Shaanxi, 710032, P. R. China.; 4 School of Chemistry and Chemical Engineering, Northwestern Polytechnical University, Xi’an, Shaanxi,710129, P. R. China.; 5 Department of Pediatrics, Tangdu Hospital, The Fourth Medical University, Xi’an, Shaanxi, 710038, P. R. China.

**Keywords:** asymmetric bioadhesive patches, sutureless sealing, soft tissue injuries, postoperative adhesion, wet interfacial adhesion

## Abstract

**Rationale:**

Anastomotic leakage (AL) remains a major surgical challenge that is difficult to address using hand-sewn closure alone. Robust, rapidly acting bioadhesive patches offer a promising alternative to conventional sutures and staples. However, the development of bioadhesive patches that achieve instantaneous wet-tissue adhesion while minimizing postoperative adhesion remains challenging. Innovative material designs and interdisciplinary translational strategies are therefore needed to advance bioadhesion technologies toward clinical application.

**Methods:**

Herein, we developed a translational asymmetric bioadhesive patch (ABP) that integrates a blood-repelling, hydrophobic, HBP oil-infused anti-adhesive top surface with a bioadhesive bottom surface capable of absorbing interfacial water. The physicochemical properties, mechanical performance, wet-tissue adhesion, and hemostatic capacity of the ABP were systematically evaluated through *in vitro* and *in vivo* assays. Its therapeutic efficacy for sutureless intestinal repair was further assessed in a rat colon defect model.

**Results:**

The ABP exhibited instantaneous and robust adhesion to various biological wet tissues (~10 times stronger than FDA-approved sealant Tisseel). The patch also showed excellent stretchability, sustaining more than 1000% strain without loss of elasticity. Moreover, ABP enables rapid hemostasis in injured tissues and achieved sutureless sealing and repair of intestinal defects in a rat colon injury model. Importantly, the asymmetric design reduced postoperative adhesions and attenuated foreign body reactions.

**Conclusions:**

The proposed ABP patch provides an effective solution for intestinal defects repair by enabling immediate wet-tissue attachment, non-invasive defect closure, and durable tissue sealing without the need for device removal. By combining strong bioadhesion with an anti-adhesive outer surface, this patch offers a promising translational platform for preventing anastomotic leakage while reducing postoperative adhesions and foreign body responses.

## Introduction

Anastomotic leakage (AL) represents the most dreaded and life-threatening complication in gastrointestinal (GI) surgery, with an incidence of approximately 10% and an associated mortality rate exceeding 30% 1, 2. Conventional stitching-and-stapling procedures, including stapled, hand-sewn, and sutureless anastomosis techniques, are essential in GI surgery. Although hand-sewn closure reinforced with fibrin sealants or other surgical adhesives is commonly used for sealing and repairing GI defects, these approaches remain insufficient to reliably prevent leakage. In high-risk patients, the incidence of AL can reach 20%, and leakage associated with sutured anastomoses may lead to severe or even fatal outcomes. Identification of risk factors is therefore essential for AL prevention 3. However, beyond risk stratification, advanced sealing technologies that can provide rapid, durable, and mechanically compliant closure of GI defects are urgently needed.

To limit leakage, tissue adhesives and sealants have emerged as promising alternatives to traditional sutures and staples for tissue repair and wound dressing across diverse surgical applications 4-6. However, the physiological environment imposes multiple biological and mechanical challenges on bioadhesive materials including adhesion under wet conditions, adapting to tissue dynamics, modulation of immune responses, mechanical matching with soft tissues, and controlled biodegradation 7. In particular, the presence of a hydration layer on the tissue surfaces is a primary cause of impaired adhesion and subsequent adhesive failure. Efficient removal or displacement of interfacial water is therefore essential for achieving reliable wet-state adhesion. Inspired by the rapid self-aggregation of hydrophobic chains into coacervate-like structures, extensive efforts have been devoted to overcoming interfacial water barrier and enhancing cohesive strength to achieve robust bioadhesion 8, 9. Nevertheless, many existing adhesives rapidly degrade upon exposure to intestinal fluids, often within hours, thereby limiting their ability to provide sustained sealing against leakage. Despite the high incidence and clinical severity of AL, reliable strategies for durable anastomotic sealing remain lacking 10. Therefore, an easy-to-use, slowly biodegradable adhesive patch capable of supporting sutureless intestinal repair is highly desirable. However, most adhesives exhibited double-sided adhesion properties, which can lead to undesired tissue adhesion to surrounding organs post-surgery.

Peritoneal adhesions (PAs) represent a serious complication of abdominal surgery 11. Following abdominal surgery, up to 54% of patients develop PAs, which is associated with chronic pelvic pain, bowel obstruction, abnormal organ function, and multiple severe complications 12, 13. Although various adhesion barriers have been developed to reduce postoperative adhesions, foreign body reactions can enhance inflammatory cell infiltration and promote long-term fibrous capsule formation at the adhesive implant-tissue interface 14, 15. In addition, non-specific protein adsorption on conventional hydrogel surfaces may increase cell adhesion and fibrosis, thereby compromising their anti-adhesion performance 14. Thus, an ideal material for GI defect repair should not only achieve rapid and durable sealing under wet and mechanically dynamic conditions but also prevent postoperative tissue adhesions and foreign body responses.

To alleviate the formation of postoperative adhesions at the implant-tissue interface, various asymmetric adhesion physical barrier system and porous adhesive hydrogels, such as DST 16, J-TP 17, PNAAA hydrogel 18, ATGel 19, AJH Patch 20, CGC 21, SAJG 22, and A-SIS bio-patch 23 have been explored in AL, tissue sealing, and wound healing. These materials provide promising strategies for leakage control and tissue regeneration. Current wet-adhesion strategies mainly rely on intermolecular interactions, polymer entanglement, mechanical interlocking, and swelling-mediated adhesion of hydrophilic bioadhesives, all of which substantially influence overall adhesive performance 5, 11, 24-26. Nevertheless, despite recent advances, barriers to clinical translation for suture repair and post-surgical adhesions still exist due to insufficient adhesion strength, delayed adherence, rapidly decayed mechanical, short degradation times, excessive swelling, non-flexible mechanical strength of internal organs and a foreign body response 27. Given the persistent clinical challenges of AL and postoperative adhesions in soft-tissue repair, novel asymmetric bioadhesive patches are needed to simultaneously provide strong tissue adhesion and anti-adhesive protection against adhesion formation. Adhesives designed for sutureless anastomosis and fluid sealing must combine resistance to digestive fluids with multiple adhesion mechanisms, especially strong tissue anchoring under chemically harsh conditions. For intestinal sealing applications, desirable adhesive performance includes high interfacial toughness, burst pressure resistance, and durable wet adhesion, with reported benchmarks including interfacial toughness greater than 50 J/m², burst pressure above 120 mmHg, and wet-tissue adhesive strength exceeding 10 kPa for more than 7 days 28. These properties are critical for withstanding GI peristalsis, intraluminal fluid pressure, and tissue deformation during the healing process 28-30. Such stringent requirements have driven the rational design of hydrogel network structures and their underlying adhesion mechanisms. Ultimately, the successful translation of asymmetric bioadhesive patch technologies depends on the integrated optimization of sustainability, manufacturability, biocompatibility, functional performance, and clinical usability 31.

**Figure 1:**
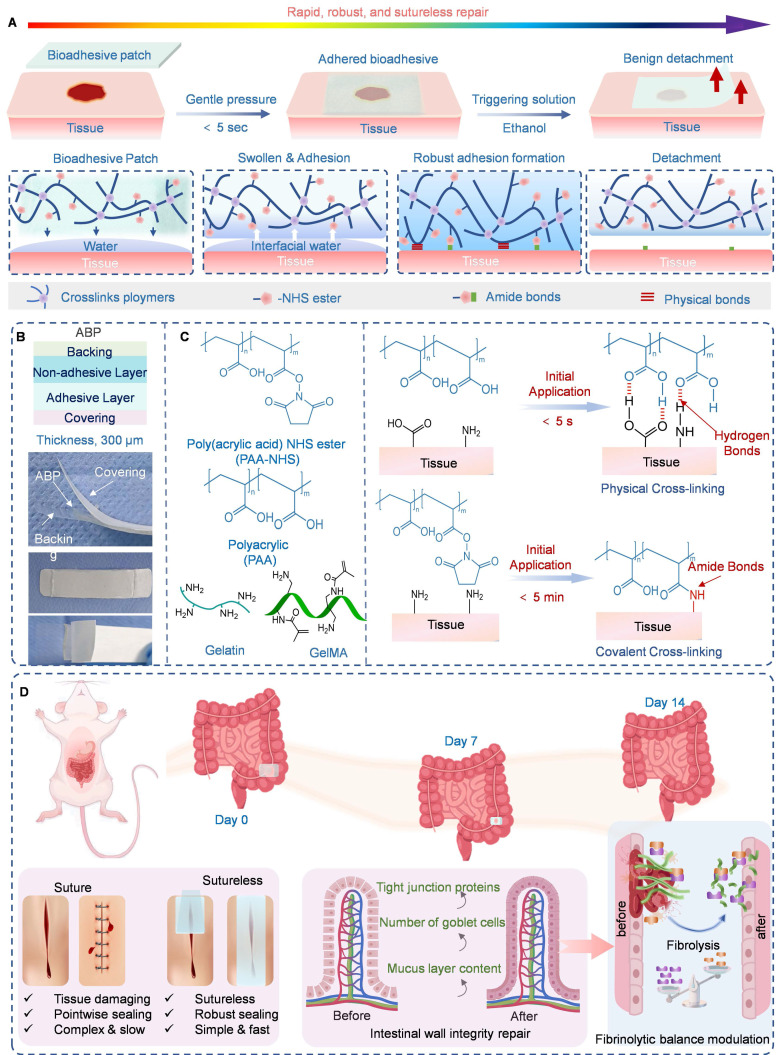
** Design and mechanism of the ABP patch for asymmetric adhesion, seamless seal, and non-pressing hemostasis. A)** Schematic illustrations depicting the rapid wet asymmetric tissue adhesion of the ABP for the sutureless sealing and repair of the intestinal defects via the rapid hydration, swelling, forming instant physical cross-linking, covalent cross-linking, and ethanol-triggerable detachment. **B)** Schematic illustration for ABP structure consisting of the non-adhesive top layer and the bioadhesive bottom layer, and the photograph of the ABP. **C)** Chemical composition and interactions of the ABP patch including hydrogen bonding (-COOH with tissue -CO-NH-, -COOH, -NH₂), electrostatic attraction (-COO^-^ with -NH₃^+^), and covalent crosslinking (-NHS with -NH₂). **D)** Mechanism illustration of colon defect repair in rat model by the ABP patch.

Herein, we developed an asymmetric bioadhesive patch (ABP) for sutureless repair of intestinal defects while preventing postoperative tissue adhesion and inflammatory infiltration. The ABP is composed of a non-adhesive hydrophobic upper layer and a robust bioadhesive lower layer with hydrophilic and hygroscopic properties. This design enables a thin, flexible, transparent, and off-the-shelf patch that rapidly adheres to diverse wet living tissues through its hydrophilic adhesive interface, while hydrophobic hyperbranched polyethylene (HBP) surface at the top layer forms a spontaneous anti-adhesive, swelling-resistant barrier to prevent peritoneal adhesion. The ABP enables instantaneous, nondestructive fixation to host tissues through interfacial adhesion (**Figure 1**). Mechanistically, the hydrophilic and hygroscopic adhesive layer rapidly absorbs interfacial water and establishes hydrogen bonding within 10 s, followed by covalent crosslinking with tissue through amide bond formation. Possessing mechanical properties comparable to abdominal soft tissues, the swollen patch withstands burst pressures greater than 56 kPa. In a rat intestinal leakage model established using large circular colonic lesions, the ABP achieved effective sealing and non-invasive repair of intestinal defects, while suppressing fibrous capsule formation compared with conventional suturing. Moreover, the triggerable benign detachment and re-adhesion capability of the ABP provides a practical strategy for non-invasive removal or repositioning. Collectively, this asymmetric bioadhesive design offers a promising translational platform for durable intestinal defect sealing, AL prevention, and postoperative adhesion reduction.

## Materials and Methods

### Materials

Acrylic acid (AAc), Acrylic acid N-hydroxysuccinimide ester (AAc-NHS ester, ≥ 90%, for peptide synthesis) were purchased by MACKLIN. Lithium phenyl-2,4,6-trimethylbenzoylphosphinate) (LAP, > 95%) was supplied by Sigma-Aldrich. Hydrophilic PU (HydroMed D3, AdvanSource Biomaterials) was utilized to fabricate the non-adhesive layer of patches. Simulated Gastric Fluid (SGF, USP, sterile, pH=1.2), Simulated Intestinal Fluid (SIF, sterile, contains trypsin and phosphate, pH=6.8) were purchased by Shanghai Yuanye Bio-Technology Co., Ltd. Moreover, weighing paper (VWR) was autoclaved for sterilization and subsequently employed as a detachable liner for the patch. All chemicals unless otherwise mentioned and used without further purification. All animal organs used in *ex vivo* experiments were obtained from supermarket.

### Method

#### Preparation of GelMA

The preparation method of GelMA was described in previous studies 32. GelMA was analyzed with ^1^H Nucleic magnetic resonance (^1^H NMR) measurement and FTIR. FTIR and ^1^HNMR spectra of GelMA is shown in **Figure S1** and **Figure S2**, respectively.

#### Preparation of PAAN

The preparation method of PAA grafted with N-hydroxysuccinimide ester (PAAN) was described in the supporting information. The synthetic route is shown in **Figure S3.** ^1^H NMR spectra of PAAN are shown in **Figure S4.**

#### Preparation of BP and ABP

To fabricate the BP, acrylic acid (30%, w/w), N-hydroxy succinimide (NHS)-grafted polyacrylic acid (PAAN) (10%, w/w), gelatin (10%, w/w), AAc-NHS ester (1%, w/w), GelMA (0.1%, w/w), and LAP (0.2%, w/w) were dissolved in deionized water. The precursor solution was then cased onto a glass mold with spacers to control patch thickness. The BP patch was fully crosslinked via exposure to UV light (405 nm, 25 mW/cm^2^) in a chamber for 1 minute.

To prepare the ABP, a non-adhesive layer was subsequently formed on the as-prepared BP by spin-coating a solution containing hydrophilic polyurethane (PU; 10%, w/w) and hydrophobic hyperbranched polyethylene (HBP; 2%, w/w) in an ethanol/H₂O mixture (95:5, v/v), as illustrated in **Figure S5**. Weighing paper was used as a detachable liner for the ABP.

#### Characterization of hydrogels

##### FTIR, ^1^HNMR spectra, and SEM characterization

The chemical structures were evaluated by ^1^HNMR (dissolved in D_2_O, 600 MHz, AV II-400 MHz, Bruker, Switzerland) and Fourier transform infrared (FTIR) spectra (Nicolet 5700, Thermo Scientific). SEM (Inspect F, FEI, USA) was used to analyze the morphological features of the hydrogels.

##### Rheological properties

A rotational rheometer (an Anton Pear MCR-302 rheometer) was used to assess the rheological behaviors of the patches. All measurements were conducted with a cone plate rotor (CP25-3, diameter: 20 mm, angle: 1° at 37 °C). The rheometer fixture was a steel cone plate with a diameter of 15 mm and a gap of 1.0 mm. Frequency sweeps (1% strain, 0.1 and 100 rad/s, 37 °C), and Strain-dependent (ω = 10 rad/s, 37 °C) were conducted on the ABP.

##### Time-dependent water contact angle test

Time-dependent water contact angle (WCA) measurements were determined by applying 20 µL of deionized water on the sample surface to observe interfacial asymmetry at room temperature using a surface contact angle meter (POWEREACH, China).

##### Swelling behavior

To assess the swelling behavior of hydrogels, *W*_0_ of wet ABP with 15 mm diameter, 1 mm in thickness, were soaked into 10 mL of PBS, SGF, and SIF under 37 °C, respectively. At 1, 3, 7, and 14 days, the swelling behavior was observed in **Figure S13**. In addition, the ABP degraded for 14 days was freeze-dried and observed with SEM. Moreover, the swelling ratio of the ABP patch and PU/HBP backing layer were evaluated in SIF (pH = 6.8) at various durations under 37 °C, respectively. The swelling ratio was calculated using the standard formula:

Swelling Ratio (%) = (W_t_-W_0_)/W_0_×100%

Where, W_t_ and W_0_ are the weights of the swollen gel at equilibrium and of the initial gel, respectively.

##### Mechanical properties tests

The wet porcine skin was utilized as the model tissue for adhesion property analysis. Three different categories of mechanical tests were then conducted. The interfacial toughness and adhesion energy by T-peel tests, and the shear strength via lap-shear tests were measured through an electronic universal testing machine (CMT 8505) at a constant displacement velocity of 50 mm/min (according to ASTM F2256, ASTM F2255, and ASTM F2258) 5. Tissue samples were sprayed with PBS suffer to prevent degradation and dehydration. After that, the samples were incubated at 37 °C for 30 min with pressing (10 kPa) pressure applied.

All tests were executed showing a constant peeling velocity of 50 mm/min. Aluminum fixtures were employed through cyanoacrylate glues, aimed at offering grips for tensile tests. The formulas used for the calculations are as follows:

Interfacial toughness = 2*F*_plateau_/*W*

Shear strength = *F*_max_/*WL*

Which L_0_ is the original length of the sample before testing; L is the length of the sample after it has broken; *F*_max_ is the maximum force of the lap shear test; *F*_plateau_ is plateau force of the 180° peeling test, and *W* and *L* denote the width and length of the adhesive area.

##### Bursting pressure tests

Burst pressure tests were performed using porcine intestine, stomach, and skin tissues with a standardized defect measuring 10 mm in length. Each tissue sample was fixed on a cylindrical testing chamber connected to a syringe pump and a pressure monitor. The chamber was filled with N₂, and an ABP sample measuring 20 × 20 × 1 mm³ was applied to cover the defect. After 5 min of adhesion, pressure was gradually applied at a flow rate of 10 mL/min. The maximum pressure recorded immediately before failure of the seal over the defect was defined as the burst pressure.

##### *In vitro* tissue adhesive tests and sealing performances

Adequate adhesion between hydrogel adhesives and injury tissues can not only realize rapid hemostasis and prevention of body fluid leakage but also establish a favorable environment for injury healing. The adhesive characteristics of the ABP were systematically assessed across diverse porcine organs, including the heart, liver, spleen, lung, kidney, and skin. The hydrogels were subjected to different deformations, including twisting and folding, to evaluate their adhesive flexibility for various tissues.

To assess *in vitro* sealing efficacy and stability in damaged porcine viscera (intestine, stomach, and heart), 10 mm, 15 mm, and 20 mm punctures were intentionally introduced and subsequently treated with the ABP. The treated porcine intestine and stomach were filled with water, and any fluid loss was assessed. Following 2 h of sealing, direct exposure to running water was employed to visualize the sealing performance. Furthermore, the adhesion interface between the ABP and tissues was examined using SEM analysis.

##### *In vitro* biocompatibility

The LIVE/DEAD assay was utilized for evaluating the biocompatibility of the ABP patch in Caco-2, a human intestinal epithelial cell line *ex vivo*. Detailed protocols of the *in vitro* biocompatibility experiments were based on previous reports 4. The cell viability was determined by the following formula:

Cell Viability (%) = (A_1_-A_2_)/(A_1_-A_0_)×100%

Where, A_0_, A_1_, and A_2_ are the absorbance values of the solvent control, normal culture control, and experimental group, respectively.

##### *In vitro* scratch assay

A scratch assay was done to investigate cell migration capacity. Briefly, after seeding cells in dishes, a scratch was introduced into the monolayer cell with a 1000 µL pipette tip. Images of the scratch were acquired at 0 h, 12 h, and 24 h. Relative wound area (%) at each time point (t) was calculated using the following formula:

Relative Wound Area (%) = (A_t_-A_0_)/A_0_×100%

Where: A_0_ is the initial wound area measured immediately after creating the scratch (at time 0 h). A_t_ is the remaining wound area at 12 h or 24 h. Quantitative results from three independent biological replicates were subjected to statistical analysis.

##### *In vivo* hemostatic ability of hydrogels on rat liver and tail

To evaluate the *in vivo* hemostatic efficacy of the ABP, Sprague-Dawley rats weighing 225-250 g and aged 6-8 weeks, including both males and females, were used for animal studies. A rat liver incision bleeding model was established to assess the hemostatic performance of the ABP in the absence of anticoagulation 33. After anesthesia with 1% sodium pentobarbital at 40 mg/kg via intraperitoneal injection, laparotomy was performed to expose the liver. A defect measuring 5 mm in length and 3 mm in depth was created in the left hepatic lobe, resulting in visible bleeding. The ABP or commercial adhesives, including Tisseel and Coseal, were then applied in situ to the wound site with constant pressure for 2 min to ensure proper adherence and bleeding control. The hemostatic time was recorded, and total blood loss was quantified by weighing pre-weighed absorbent papers before and after blood absorption. Untreated wounds served as blank controls. The hemostatic properties of the ABP were further evaluated using a rat tail transection model. After anesthesia, the rat tail was transected 4 cm from the base and immediately placed onto pre-weighed filter paper. The ABP was applied to the bleeding site, and blood loss was determined by measuring the increase in filter paper weight. Each experimental group included four rats.

#### *In vivo* rat abdominal skin model for subcutaneous implantation

Six-week-old rats (200-250 g) received a 15-mm midline abdominal incision followed by laparotomy to expose the abdominal wall. Sterilized ABP and BP adhesive (10 mm in diameter and 1 mm in thickness) implants were subcutaneously implanted to the abdominal wall of rats (n = 3 per group). the muscle and skin incisions were closed with sutures. At Day-7 and Day-14 post-implantation, the residual patch and skin tissue were collected and fixed for histological and immunofluorescence.

#### *In vivo* colon defect repair in the rat model

The *in vivo* colon defect repair model was established in rats as previously reported 4. Rats (male, Sprague Dawley, 200-230 g) were fasted for 24 h before surgery to empty the descending colon. Under anesthesia, the rats were placed in the dorsal recumbent position, and the abdomen was shaved and aseptically prepared. A midline abdominal incision was made, and the colon was carefully exteriorized. A 5-mm full-thickness defect was created in the colon to mimic an anastomotic leak. The ABP was then positioned over the defect after ethanol application to temporarily reduce adhesion. After lavage, the colon was returned into the abdominal cavity, and the abdominal incision was closed. For long-term assessment, rats were euthanized at Day-14 post-surgery. The patched wound tissues were harvested and fixed for histological and immunofluorescence analyses.

#### Statistical analysis

One-way ANOVA with Tukey’s post hoc test was used for multiple comparisons. The threshold for significance was set at *P ≤ 0.05, **P ≤ 0.01, ***P ≤ 0.001, and ****P ≤ 0.0001.

## Results and Discussion

### Design and mechanisms of the ABP

The key requirement for achieving robust wet-tissue adhesion while preventing post-surgical adhesions are disruption of the interfacial hydration layer and establishment of strong tissue-material interactions 34, 35. To address these requirements, we fabricated an asymmetric bioadhesive patch (ABP) designed for sutureless intestinal defect repair, postoperative adhesion prevention, and attenuation of inflammatory infiltration (**Figure 1A**). The ABP integrates a non-adhesive hydrophobic top layer composed of hydrophobic polyurethane (PU) and hydrophobic hyperbranched polyethylene (HBP) with a hydrophilic, hygroscopic and robust bioadhesive bottom layer, resulting in a thin, flexible, and transparent patch with mechanical properties well matched biological tissues (**Figure 1B**).

The bioadhesive bottom layer is composed of an interpenetrating crosslinked network of PAA, and PAA grafted with N-hydroxysuccinimide ester (PAAN), GelMA, and gelatin. Upon contact with moist tissue surfaces, the layer rapidly absorbs interfacial water and physiological fluids within 5 s, promoting hydration, swelling, and immediate physical crosslinking at the tissue-patch interface. Under mild pressure of approximately 1 kPa, the adhesive layer instantaneously forms hydrogen bonds and electrostatic interactions with tissue, followed by covalent crosslinking through amide bond formation.

Mechanistically, the hydrophilic PAA polymer network within the bioadhesive layer provides abundant carboxyl groups (-COOH), which form instant physical crosslinks, mainly with hydrogen bonds, with amide bonds (-CO-NH-) and amino groups (-NH_2_) on tissue surface. Meanwhile, negatively charged carboxylate groups (-COO^-^) in the PAAN network further promote interfacial physical crosslinking through hydrogen bonding (X-H···Y) and electrostatic interactions with protonated amino groups (-NH₃⁺) on tissues, enabling robust adhesion within several seconds 36. Subsequently, the adhesion interface is further enhanced through covalent cross-linking, as -NHS ester groups in the PAAN network react with tissue amino groups to form stable amide bonds within minutes, thereby enabling effective tissue sealing without requiring additional sustained pressure (**Figure 1C**) 4, 16.

In contrast, the hydrophobic HBP-containing top layer functions as a non-adhesive, swelling-resistant barrier. This hydrophobic surface prevents undesired tissue attachment on the outward-facing side of the patch and thereby helps reduce peritoneal adhesion formation (**Figure 1D**). Moreover, the interfacial adhesion generated by the ABP can be rapidly reversed within 5 s by applying an ethanol-triggered solution, enabling on-demand, atraumatic detachment from the adhered tissue 37. Together, this asymmetric design enables rapid wet-tissue anchoring, durable sealing, anti-adhesive protection, and controllable detachment, supporting its potential use for sutureless intestinal repair.

**Figure 2:**
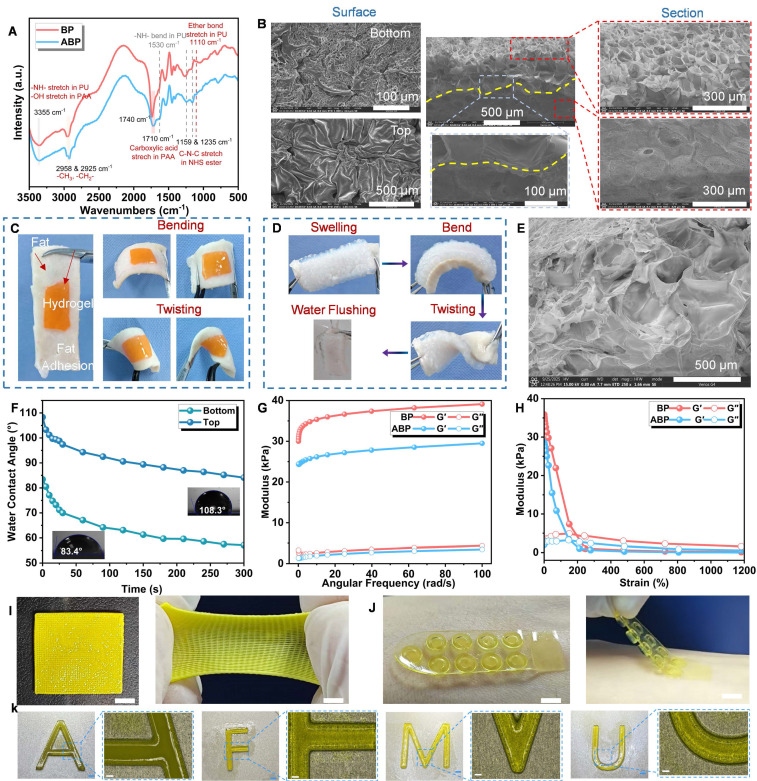
** Characterization of the asymmetric mechanism for the ABP hydrogel. A)** FT IR spectra of the BP and the ABP. **B)** SEM images of the surface and section morphology for the ABP. **C)** Images of the ABP adhered to porcine fat under bending and twisting. **D)** Images of the ABP adhered to porcine skin tissue under various mechanical conditions, including bending, twisting, and water flushing after being swollen in H_2_O for 14 days. **E)** SEM images of the ABP bioadhesive after 14 days in SIF.** F)** The time-dependent water contact angle tests for the bottom and top surfaces of ABP hydrogel to evaluate their hydrophilicity, respectively. **G)** Frequency-dependent (γ= 1%, 37 °C) oscillatory shear rheology and **H)** Strain-dependent (ω = 10 rad/s, 37 °C) of the ABP. **I)** 3D printed adhesive patch with mechanical toughness. **J)** 3D printed octopi-inspired hydrogel suckers with tissue adhesion. Scale bars, 1 cm (i to j). **K)** Optical micrographs of 3D printed letter patterns (“A”, “F”, “M”, “U”), showing micrometer-scale resolution (line width ~500 µm, layer thickness 100 µm). Blue scale bar: 500 μm, white scale bar: 200 μm.

The chemical structures of the BP and ABP were verified by FTIR spectroscopy. As shown in **Figure 2A**, the symmetric and asymmetric stretches of C-N-C at 1159 cm^-1^ and 1235 cm^-1^ are associated with PAAN in the ABP 4, 38. The stretching vibrations of the C=O and O-H bonds in -COOH are detected at 1710 cm^-1^ and 3350 cm^-1^ in PAA, confirming the successful incorporation of PAA *via* grafting and physical entanglement. Compared to BP patch without a non-adhesive top layer, the stretching and bending vibration bands of the -NH- bond in the O=C-NH-C linkage are identified at 3355 cm^-1^ and 1530 cm^-1^ in PU-containing spectra, confirming the existence of a urethane functional group. Specifically, the strong stretching vibration band corresponding to the ether bond at 1100 cm^-1^ reflects the polyether nature of the ether-based PU 39. These FTIR results confirmed the successful fabrication of the ABP. SEM was then used to examine the microstructure of the ABP. As shown in **Figure 2B**, ABP exhibited a typical asymmetric architecture. The bottom surface exhibited a distinct interconnected porous structure, which can prevent crack propagation, facilitate water transport within the hydrogel, and enhance water absorption capacity 40. In contrast, the top surface showed a denser interlinking 3D microstructure with some lamellar surface features.

The ABP further exhibited excellent adhesion to wet porcine fat and skin tissue under various mechanical conditions, including bending and twisting. Remarkably, the ABP maintained robust wet-tissue adhesion and withstood sustained bending, twisting, and large strain stretching without fracture even after 14 days of swelling in SIF (**Figure 2C-D**). These favorable mechanical properties are essential to realizing tough and durable bioadhesion. As shown in **Figure 2E,** enlargement of the hydrogel microporous structure after 14 days of swelling was associated with its degradation behavior.

To evaluate the hydrophobic top layer, time-dependent contact angle measurements were performed (**Figure 2F**) 41. Although both surfaces contained hydrophilic components, the WCA of the top surface remained consistently higher than that of the bottom surface at all time points, indicating the asymmetric distribution of hydrophobic HBP components and hydrophilic -COOH groups. Specifically, the ABP exhibited a WCA of 108.3° on the top surface and 83.4° on the bottom surface, confirming that the hydrophobic HBP-containing layer was mainly located on the top side, whereas the hydrophilic -COOH-rich adhesive layer was exposed on the bottom side 42. The antifouling behavior of the two surfaces was further compared by exposing the non-adhesive hydrophobic top layer and the bioadhesive bottom layer to porcine blood. When submerged in a porcine blood bath, the bioadhesive layer was immediately wetted by blood, whereas the non-adhesive hydrophobic top layer resisted blood contamination and remained intact due to the protective HBP-containing surface (**Figure S6**) 43. These results demonstrate that the ABP possesses distinct wettability and bioadhesive properties on its two sides, the hydrophilic hydrogel side exhibits strong tissue adhesion (**Figure S7**), whereas the PU/HBP-infused side provides water-repellent and anti-adhesive effects.

Rheological analysis further confirmed the stability of the hydrogel network. As shown in **Figure 2G**, the storage modulus (G’) was consistently higher than the loss modulus (G”), indicating that the dynamic hydrogel network remained stable and behaved predominantly as an elastic solid 44. Compared with ABP, BP exhibited higher G′ values, which may be attributed to differences in network composition, crosslinking density, and the absence of the non-adhesive top layer. In the strain sweep test, the G′ values of both BP and ABP decreased rapidly with increasing strain at 37 °C, and the crossover points of G′ and G″ occurred at strains above 200% and 150%, respectively, indicating disruption of the hydrogel elastic network (**Figure 2H**). The broad strain-tolerant region suggests that the hydrogels can maintain structural integrity under the dynamic mechanical environment of the body.

The incorporation of energy-dissipating and self-healing mechanisms is important for designing tough, stretchable, and self-recoverable adhesive materials. To assess self-healing behavior, the ABP was manually cut with a scalpel. After recovery at 37 °C for 1 h, the two separated pieces rejoined and restored their original structure, likely through re-crosslinking of the PAA-based network. The healed sample withstood bending, twisting, and large-strain stretching without fracture (**Figure S8**). This efficient self-healing behavior may facilitate interlayer integration in subsequently printed structures. The dynamic behavior of ABP is mainly attributed to reversible disruption and reconstruction of non-covalent crosslinks rather than irreversible fracture of covalent polymer networks.

Finally, an all-in-one adhesive patch was fabricated via high resolution desktop projection-based 3D bioprinter (EFL-BP-8601Pro) based on digital light processing (DLP). This precise printing enables the fabrication of tissue-adhesive patches with controlled sizes and structural asymmetry, which demonstrated excellent tensile strength and mechanical toughness in **Figure 2I**. Furthermore, DLP 3D printing of octopi-inspired hydrogel sucker can adhere to the skin tissue in **Figure 2J-K**. Together, this printable precursor solution and high-resolution DLP printing platform enable the fabrication of size-controlled bio-bandages, bio-tapes, patches, and complex octopus-inspired adhesive structures at micro- and millimeter scales.

### Mechanism and tissue tough adhesive performance

**Figure 3:**
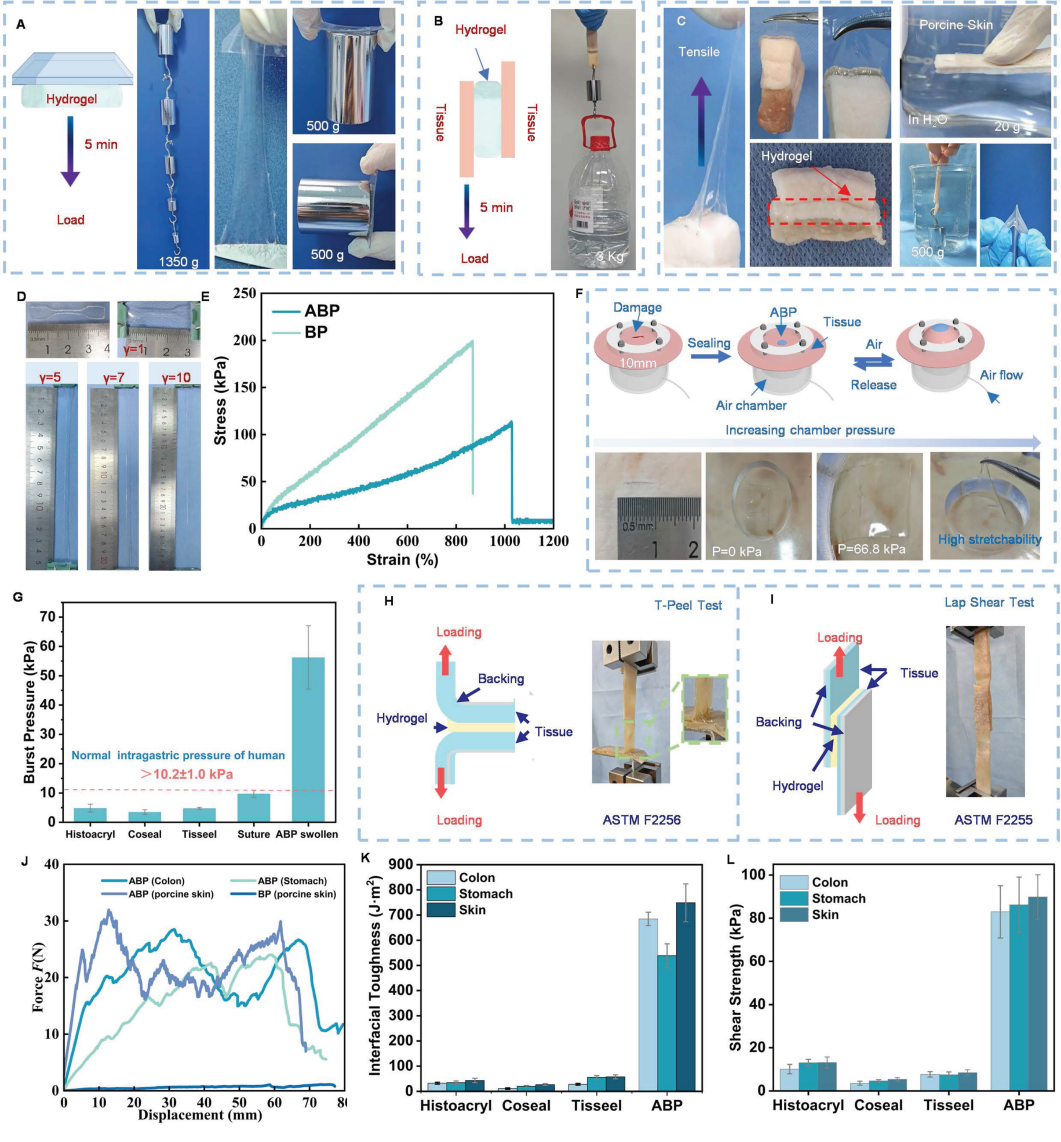
** Mechanical and tough adhesion performance of the rapid asymmetric bioadhesive patch ABP. A)** Photographs of the ABP with a 20 mm diameter and 1.0 mm thickness adhered to the surface between stainless steel and glass, carrying 1350 g of weight. **B)** Photographs of the ABP with a 20 mm×20 mm×10 mm adhered to the tissue between two pieces of porcine skin carrying 3 Kg of weight. **C)** Photos of ABP adhesion to and detachment from the porin fat layer, the ABP patch bearing a 500 g weight under H_2_O, and a sharp stimulus being applied to the ABP. **D)** Images of the ABP stretched to more than 10 times the original length. **E)** Typical stress-strain curves of BP and ABP.** F)** Assessment of bursting pressure utilizing ABP and commercial tissue adhesives on moist porcine skin, intestine, and stomach tissue. **G)** Results of the maximum burst pressure of *in vitro* porcine colons, stomach, and skin with a 10-mm-diameter defect sealed by the ABP patch, commercial tissue adhesives (n = 3). **H)** Schematic of the T-peeling test for interfacial toughness measurements following ASTM F2256). **I)** Schematic of the lap-shear test for shear strength measurement following ASTM F2255. **J)** Representative force/width *vs*. displacement curves form the 180-degree peel tests. Interfacial toughness **K)**, and shear strength **L)** of the ABP and commercial tissue adhesives.

Robust, rapid, and durable adhesion is essential for maintaining effective sealing under tissue deformation, fluid exposure, and high-pressure physiological conditions. As shown in **Figure 3A**, the ABP rapidly adhered to stainless steel and glass, and was able to lift a 1350 g weight, demonstrating strong adhesion to non-biological substrates. The ABP also firmly adhered between two pieces of porcine skin and supported a 3 kg weight without detachment (**Figure 3B,** & **Video S1**). Moreover, the ABP rapidly adhered to the fat layer, and lift a 500 g submerged block out from water without adhesive failure of (**Figure 3C**), further demonstrating its strong wet-adhesion capacity.

The ABP also exhibited excellent stretchability. As shown in **Figure 3D**, the ABP could be stretched to more than ten times its original length. Compared with BP, the strain at break of the ABP patch increased markedly from 750% to 1020%, which may be attributed to the excellent ductility of the PU top layer and the formation of an interpenetrating double-network structure (**Figure 3E**). The bottom bioadhesive layer further enabled conformal and stable integration with wet tissues. Together, these structural features allowed the ABP to withstand large deformation and strain, supporting its fracture-resistant performance under intended use conditions.

The adhesive performance of the ABP was characterized by measuring interfacial toughness, shear strength, and burst strength using wet *ex vivo* porcine tissue (**Figure 3F** & **3G-I**). After 5 min of adhesion, the ABP achieved a burst pressure of 56.1 kPa, corresponding to approximately 420 mmHg. This value was approximately three to four times higher than normal human systolic blood pressure and markedly exceeded normal physiological intragastric pressure, as well as the sealing capacity achieved by surgical sutures in the present model (**Figure 3G**). Specifically, suture-treated porcine stomach exhibited a mean burst pressure of approximately 9.7 kPa (72.8 mmHg). In comparison, commercial tissue adhesives, including Histoacryl, Coseal, Tisseel, Suture, and tissue adhesives, showed substantially lower burst pressure values (<5 kPa) (e.g., less than 5.6 ± 0.5 mmHg for stomach tissue) indicating insufficient sealing performance under physiologically relevant pressure conditions. These results demonstrate that the ABP provides superior sealing performance across diverse tissues and outperforms existing commercial tissue adhesives under challenging wet, pressurized, and fluid-flow conditions 45, 46.

When applied to wet tissues under gentle pressure of approximately 1 kPa, the ABP exhibited strong adhesive performance within 5 min. The interfacial toughness reached 685.7 J/m² for colon, 539.5 J/m² for stomach, and 748.8 J/m² for skin. Similarly, the shear strength reached 82.5 kPa for colon, 85.9 kPa for stomach, and 89.7 kPa for skin. These values were higher than those of commercial tissue adhesives, including Histoacryl, Coseal, and Tisseel (**Figure 3J,** &** Figure 3K-L**). On wet tissue surfaces, the hydration layer is a major barrier that impairs adhesive contact. Therefore, efficient removal or displacement of this interfacial water layer is essential for achieving robust wet-state adhesion. In the ABP, the PAA polymer networks rapidly absorbing interfacial water enhance bioadhesion through and promotes hydrogen bonding and electrostatic interactions within seconds. Subsequently, the adhesion was further enhanced through the formation of multiple covalent bonds between the adhesive layer and tissue surface. In addition, the high toughness of the hydrogel enables substantial energy dissipation under mechanical loading, contributing to durable tissue sealing.

The ABP also demonstrated robust adhesion to diverse wet tissues, including heart, colon, and liver (**Figure S9-10**). Even after being subjected to vigorous water spraying, the ABP remained firmly adhered to liver tissues (**Figure S11**), and continuous peeling led to rupture of the porcine liver rather than adhesive detachment. This strong interfacial bonding further supports the robust adhesive performance of the ABP. Notably, the ABP retained its ability to form strong adhesion to wet tissues after storage for more than 14 days 5. Additional demonstrations showed that the BP could lift 1250 g weights and a 185 g glass reagent bottle, and could adhere to and detach from stainless steel, glass, and PVC surface (**Figure S12**). Compared with previously reported bioadhesives, the mechanical toughness and wet-tissue adhesive performance of the ABP were comparable or superior (**Table S1**). Although injectable adhesives such as Tisseel and Coseal and patch-type adhesives differ in application mechanics, direct head-to-head comparison with leading commercial adhesive patches would be valuable in future studies.

Swelling of hydrophilic bioadhesives is an effective wet-adhesion strategy because it promotes interfacial contact, compresses surrounding tissues, and facilitates rapid intermolecular bond formation 5, 24. To evaluate the swelling behaviors *in vitro*, ABP samples with a diameter of 15 mm were immersed in well plates at 37 °C in H_2_O, PBS, SIF (pH 6.8), SGF (pH 1.2), and the media were replaced at predetermined time points. The bioadhesive bottom layer exhibits pH-responsive rapid hydration, swelling, and rapid physical cross-linking behavior on wet tissue by absorbing native physiological fluids and/or moisture (**Figure S13**). In particular, the ABP preferentially absorbed intestinal fluid over gastric fluid, suggesting its potential to provide a first-line sealing barrier in the event of an anastomotic leak. This distinct swelling behavior may be attributed to protonation of carboxyl groups in the PAA chains under acidic SGF conditions, which reduces interchain electrostatic repulsion, promotes hydrogen bond formation, and thereby decreases the swelling ratio 47. Importantly, integration with the hydrophobic PU/HBP layer substantially restricted excessive, ensuring mechanical stability during clinical application (**Figure S14**). Moreover, the adhesive performance of ABP was largely maintained for 14 days in SGF, which is critical for sustaining stable contact between the hydrogel and tissue during the healing period (**Figure S15**).

### *In vitro* biocompatibility and *in vivo* degradation

**Figure 4:**
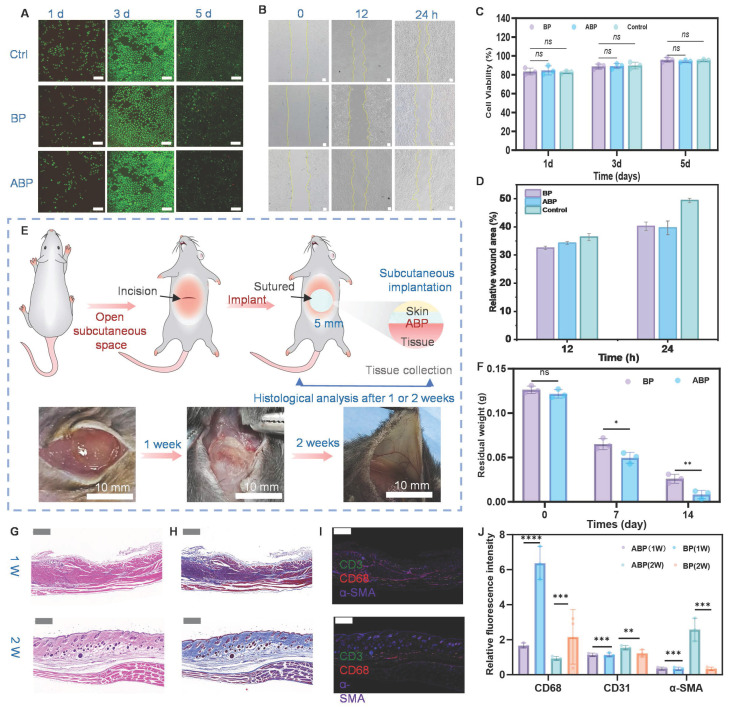
***In vitro* biocompatibility and *in vivo* biodegradability of the GI patch within a rat subcutaneous model. A)** Representative LIVE/DEAD assay images and **C)** CCK-8 assay cell viability of Caco-2 for control (DMEM), the BP and the ABP with and without ethanol treatment after 1, 3, and 5 days of culture, Scale bar = 200 μm. **B)** Microscopic images and **D)** Relative wound area of the scratch assay of Caco-2 cells for control, the BP and ABP after 0 h, 12 h and 24 h (n = 3). Scale bar: 100 μm. **E)** Schematic diagram of the subcutaneous implantation of the ABP on the rats′ abdominal skin. Images of ABP hydrogels after implantation for 1 week and 2 weeks. **F)** Residual weight of ABP following subcutaneous implantation in rat abdominal skin (n =3). **G)** Representative H&E histological images and **H)** Masson staining images around the ABP after subcutaneous implantation for 1 week and 2 weeks. **I)** Representative images of CD31, CD68, and α-SMA immunostaining in wound tissue at 1 week and 2 weeks. **J)** Relative fluorescence intensity of CD31, CD68, and α-SMA-positive signals for the subcutaneously implanted ABP patch and BP patch at 1 week and 2 weeks following implantation. Scale bars, 0.5 mm.

Biocompatibility and degradability are essential requirements for tissue adhesive patches for internal use. To quantitatively evaluate the biocompatibility and degradation profile of the ABP, a series of *in vitro* and *in vivo* characterizations were performed. LIVE/DEAD staining of Caco-2 cells cultured with ABP-conditioned medium for 1, 3, and 5 days showed cell viability comparable to that of the control medium group, indicating favorable cytocompatibility (**Figure 4A & Figure 4C**). Scratch wound assays were further conducted to assess the effect of ABP-conditioned medium on epithelial cell migration. As shown in **Figure 4B**, efficient Caco-2 migration was observed at 12 h and 24 h. Quantitative analysis showed that at 24 h, the relative wound areas in the BP-treated (41% ± 1.3%) and ABP-treated group (40% ± 2.1%) were slightly lower than that of the control group (49.2% ± 1.2%) (**Figure 4D**), suggesting that the BP and ABP did not impair epithelial cell migration.

To evaluate *in vivo* degradation and tissue compatibility, ABP samples were subcutaneously implanted into the abdominal region of rats and analyzed by gross observation, H&E staining, Masson’s trichrome staining, and immunofluorescence. The ABP gradually decreased in size over time and functioned as a physical barrier to inhibit postoperative fibrotic adhesions to the surrounding tissues. Bulk hydrogel remnants were retained subcutaneously at Day-7, whereas no visible hydrogel fragments were embedded in the surrounding tissues by Day-14 post-implantation (**Figure 4E-F**, & **S16**). Compared to the BP control group, the residual weight of ABP following subcutaneous implantation was significantly reduced, indicating near-complete degradation. These results demonstrate the favorable *in vivo* degradability of the ABP.

H&E staining further confirmed that the subcutaneously implanted ABP degraded within 2 weeks and showed no evident tissue adhesion at the ABP-tissue interface (**Figure 4G**). Masson’s trichrome staining was used to evaluate collagen deposition and tissue remodeling during wound healing. The ABP group exhibited enhanced collagen deposition (**Figure 4H**), suggesting favorable tissue repair and pro-healing effects in soft-tissue defects. During the degradation period, the ABP persisted at the subcutaneous implantation site for approximately 14 days and induced minimal inflammation and fibrous capsule formation in surrounding tissues. This degradation profile is beneficial for suppressing adhesion formation. In contrast, severe adhesions were observed in the control group 2 weeks after surgery, accompanied by acute inflammatory cell infiltration and tissue necrosis around the defect area. These findings confirm the anti-adhesion advantage of the asymmetric patch structure.

Immune and stromal cell infiltration at the implant-tissue interface was further assess by immunofluorescence staining for fibroblasts markers (αSMA), T-cell markers (CD31), and macrophage markers (CD68) (**Figure 4I**). Compared with control groups, ABP-treated tissues showed increased expression of α-SMA and CD31 (**Figure 4J**), indicating active tissue remodeling and angiogenesis during wound healing 48. In contrast, CD68 expression was markedly lower in the ABP group than that of the controls, suggesting reduced macrophage infiltration and attenuated inflammatory response. Since excessive or persistent inflammation often contributes to impaired wound healing and fibrotic adhesion formation, these findings provide histological evidence supporting the pro-healing and anti-inflammatory performance of the ABP 49. Overall, the ABP possesses favorable biocompatibility, biodegradability, tissue compatibility, and pro-regenerative properties, supporting its potential application for *in vivo* wound repair.

### *In vivo* hemostatic performance of the ABP

**Figure 5:**
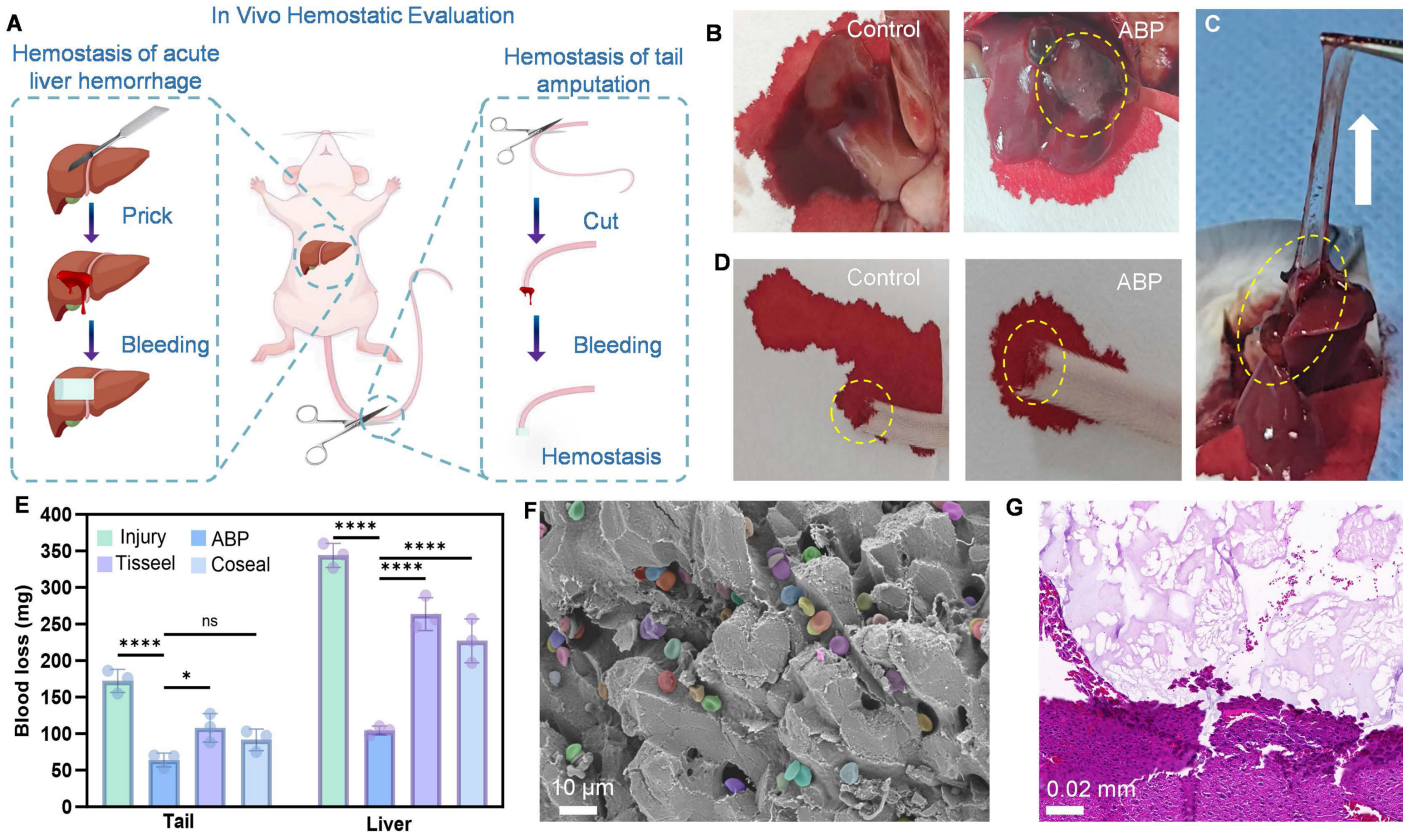
***In vivo* sutureless sealing efficacy of the ABP patch in rat models**. **A)** Schematic illustration of the hemostatic process using ABPs in a rat liver injury and tail amputation hemostasis model. Hemostatic sealing images of a bleeding rat liver **B)** and tail **D)** in the blank group and the ABPs group. **C)** ABPs robustly seal rat liver injury. **E)** Total blood loss of liver and tail hemostasis in the control group and the ABP group of rats at 120 s (*n* = 4, independent samples). Representative SEM **F)** and H&E **G)** images of blood cell adhesion to ABP surface in a model of liver hemorrhage.

Uncontrolled bleeding and hemorrhage remain major causes of morbidity and mortality during traumatic injury and surgery. Even though wet-tissue adhesives have shown promise for sealing tissue defects, their hemostatic efficacy requires systemic validation. To evaluate the *in vivo* hemostatic performance of the ABP, rat liver injury and tail transection bleeding models were established (**Figure 5A**). As shown in **Figure 5B & 5D**, continuous bleeding was observed in the untreated control group after 3 min. In contrast, the ABP robustly sealed the rat liver injury site and rapidly arrested hemorrhage (**Figure 5C**). Quantitative analysis showed that ABP treatment reduced blood loss from 340 mg in the control group to 95 mg within 3 min. Similarly, in the rat tail transection model, ABP treatment reduced blood loss from 170 mg to 60 mg within 3 min compared with the control group (**Figure 5E**). These findings indicate that the ABP can effectively mitigate severe bleeding following tissue injury 50. Compared to commercial injectable tissue adhesives including Tisseal and Coseal, the ABP demonstrated superior hemostatic performance, as characterized by the shorter hemostatic time and reduced blood loss. During the hemostatic sealing process, the bioadhesive layer rapidly absorbs blood at the wound interface, concentrating coagulation factors and blood cells, while simultaneously forming strong interfacial bonds with the tissue substrate. This process accelerates clot formation and promotes stable sealing of the bleeding site 51.

Representative SEM and H&E staining images from the rat liver hemorrhage model further confirmed the hemostatic activity and hemocompatibility of the ABP. SEM images showed pronounced blood cell adhesion and aggregation on the ABP surface (**Figure 5F**). Consistently, H&E images demonstrate a marked accumulation of blood cells at the liver injury site after ABP treatment (**Figure 5G**), indicating active participation of the ABP in the hemostatic process. Collectively, these results demonstrate that the ABP achieves efficient hemostasis by promoting blood absorption, blood cell adhesion and aggregation, coagulation factor enrichment, and stable tissue sealing.

### *Ex vivo* wet tissue adhesion performance

**Figure 6:**
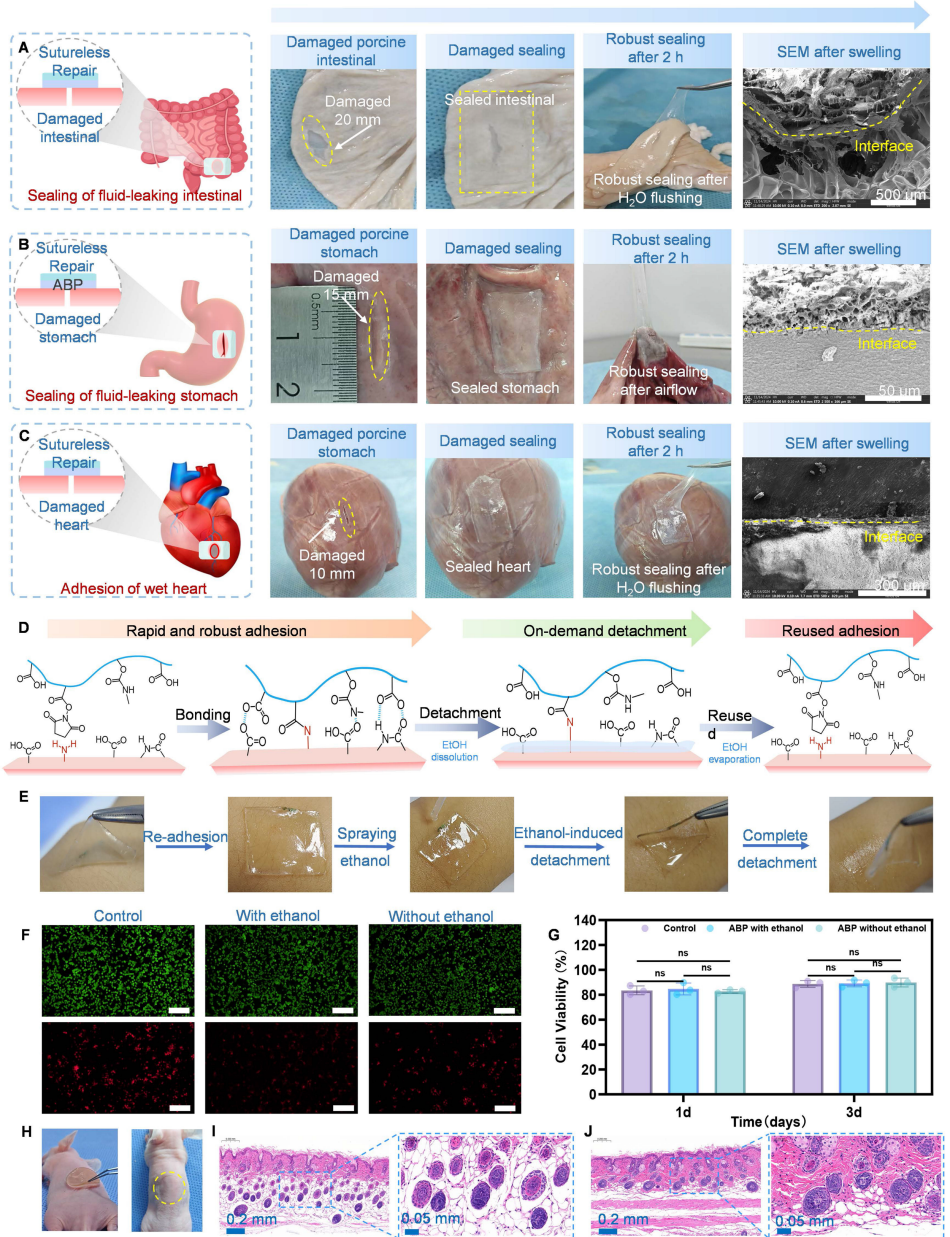
***Ex vivo* sealing and biocompatibility of the ABPs.** Illustrations, representative, and SEM images of the ABPs for *ex vivo* fluid-tight sutureless sealing of injured porcine wet intestine **A)**, a fluid-leaking porcine stomach** B)**, and PBS buffer spraying porcine wet heart **C)**, respectively. SEM of the adhesion interface between the ABP bioadhesive and porcine intestine, stomach, and heart tissues. **D)** Illustration of the adhesion, detachment, and reused adhesion mechanism by ethanol between the ABP patch and tissue. **E)** Representative peeling process of the ABP patch from human skin, after applying the ethanol. **F)** Representative LIVE/DEAD assay images and **G)** CCK-8 assay cell viability of human skin fibroblasts cells (HSF) for control (DMEM), the ABP with and without ethanol treatment, Scale bar = 200 μm. **H)** Peeling process of the ABP patch from rat skin, **I)** H&E staining images of rat skin tissues following removal of the ABP hydrogel after 24 h of adhesive, and **J)** after 5 attach/detach cycles with ethanol-induced detachment.

We performed proof-of-concept applications using *ex vivo* porcine soft-tissue defect models to evaluate the ability of the ABP to achieve instant, sutureless sealing of body fluid and air leakage 52. The ABP rapidly adhered to the damaged wet porcine intestine, fluid-leaking porcine stomach, and a PBS buffer-sprayed wet porcine heart under gentle pressure within 10 s, forming strong and durable interfacial adhesion (**Figure 6A-C**). In gastrointestinal perforation models with active fluid leakage, the ABP patch immediately sealed the damaged tissues and completely prevented excessive fluid outflow. Notably, even following 2 hours of vigorous water flushing, no secondary leakage was observed, demonstrating robust fluid-tight sealing performance. In addition, stretched ABP films remained firmly adhered to porcine intestine, stomach, and heart tissues, indicating favorable sealing capacity for soft-tissue defects exposed to internal air or liquid flow.

SEM imaging further confirmed the strong tissue integration of the ABP. As shown in **Figure 6A-C**, the ABP effectively adhered to multiple tissue surfaces and maintained a distinct two-layer asymmetric structure with a clear boundary. A conformal and continuous adhesion interface was observed between the bioadhesive layer and tissue surface, confirming intimate interfacial contact. Mechanistically, this strong and seamless adhesion may be attributed to rapid physical crosslinking, subsequent covalent crosslinking, and interfacial penetration between the adhesive and tissue surfaces 53. Taken together, these results demonstrate that the ABP enables instant and sutureless sealing of soft tissue damages under wet *ex vivo* conditions, supporting its potential as tissue adhesive patch for biomedical applications.

We then evaluated the triggerable and atraumatic detachment behavior of the ABP. Triggerable detachment of the ABP is based on disruption of physical and covalent interactions between the bioadhesive layer and the tissue surface. Ethanol was selected as a detachment trigger because of its volatility and relatively benign biological profile. Application of ethanol rapidly weakened the intermolecular interactions between the ABP and tissue surface, while also reducing the cohesive strength of the polymer network. As shown in **Figure 6D** and **Figure S17-18**, ethanol spraying markedly reduced both adhesion force and adhesion energy within approximately 5 s, converting the ABP surface from an adhesive to a non-adhesive state and enabling easy removal. After ethanol evaporation, the adhesion energy was nearly fully restored. Remarkably, this bonding-detachment-rebonding process could be repeated for five cycles without significant performance deterioration. These results indicate that the ABP can detached on demand after brief ethanol treatment and can recover its adhesive performance after ethanol evaporation. As demonstrated in **Video S2 & S3, and Figure 6E,** the ABP firmly adhered to the wet skin surface and can be cleanly detached by ethanol treatment without causing erythema or other adverse effects, demonstrating its strong wet adhesion, skin compatibility, and ethanol-triggered removability 16, 54, 55.

To further assess potential cytotoxicity after ethanol-triggered detachment, we performed cell viability assays and LIVE/DEAD staining using HSF cells cultured in extraction medium prepared from ABP patches after ethanol spraying and complete evaporation. As shown in** Figure 6F-G,** CCK-8 assay showed no significant decrease in cell viability in the ethanol-treated ABP group compared with controls treated with extraction medium from ABP patches without ethanol exposure. Moreover, after 24 h of adhesion and five ethanol-triggered attach/detach cycles, H&E staining showed that the underlying skin tissue remained intact (**Figure 6H-J**). These findings demonstrate that ethanol-treated ABP, after complete volatilization of ethanol, retains *in vitro* biocompatibility and *in vivo* tissue safety. This triggerable detachment strategy provides an on-demand, painless, and safe approach for repositioning misplaced bioadhesives and retrieving implanted adhesive devices.

### *In vivo* sutureless sealing and anti-postoperative adhesion of colon defects

**Figure 7:**
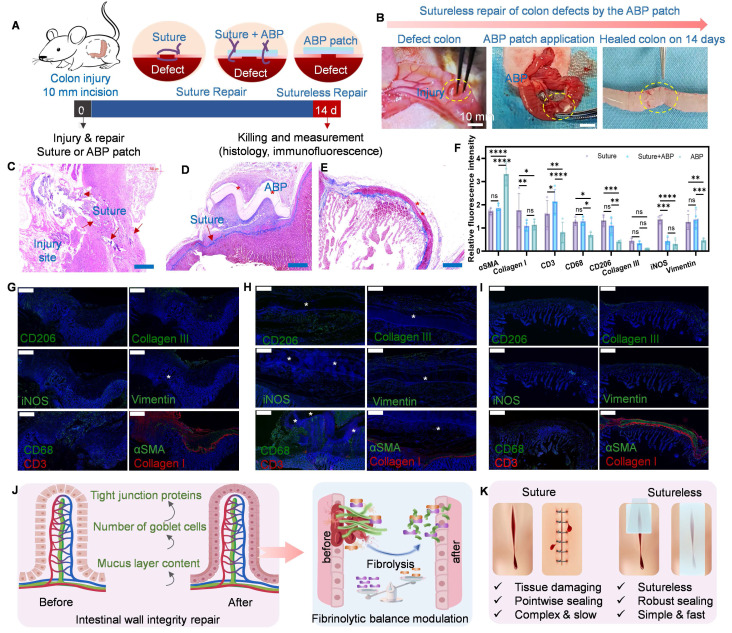
***In vivo* sutureless repair of GI defects using the ABP in the rat model. A)** Schematic and **B)** experimental process images for *in vivo* defect repair of the rat colon by sutures and the ABP. Rat colon 14 days (D14) following sutureless treatment through the ABP. Representative histological images stained with H&E for** C)** sutures only, **D)** suture with ABP, and **E)** the ABP only at 14 days (D14) after implantation in the rat colon. ^*^represents the implanted patch, scale bars, 500 μm (C to E). **F)** Relative fluorescence intensity from the immunofluorescence images for α-SMA, collagen I, collagen III, vimentin, CD3, CD68, iNOS, and CD206 at 2 weeks following implantation of sutures and the ABP. Immunofluorescence staining and normalized immunofluorescence intensity analysis of wound tissues collected at 14 days post-operation (D14). The sutures only **G)**, suture with ABP **H)**, and ABP patch only **I)** groups were used for defect repair, respectively. The results show elevated abundance of α-SMA, collagen I, and collagen III in the ABP patch group compared with the suture group. ^*^Represent the implanted sutures or ABP. Values represent the means ± SD [n = 4]. One-way analysis yielded P values; ns, not significant; ^*^P ≤ 0.05; ^**^P ≤ 0.01; ^***^P ≤ 0.001; and ^****^P ≤ 0.0001. Scale bars, 0.5 mm (G to I).** J)** Anti-adhesion effects of ABP in the rat colon defect repair model after surgery. **K)** Comparison of sutured *vs* sutureless tissue repair.

To further evaluate *in vivo* the sutureless sealing and healing performance of the ABP, a rat colon defect model was established using a 10-mm perforation, and the repaired tissues were harvested for histological analysis on Day-14 postsurgery. As shown in **Figure 7A-B**, the ABP was applied directly to the colon defect through adhesive fixation, whereas surgical suturing and suturing combined with ABP treatment served as control groups. At day 14 after implantation, no obvious congestion, redness, or inflammation was observed at the ABP-treated site during the degradation process (**Figure 7B**). Moreover, no apparent tissue adhesion was observed in rats treated with the ABP patch group, indicating a favorable defect repair and anti-postoperative adhesion efficacy 53. Overall, the rapidly adhesive ABP enabled sutureless repair for anastomotic leakage by integrating rapid hemostasis, seamless sealing, and postoperative adhesion prevention. In contrast, the control group exhibited severe fibrous adhesion between the cecal and abdominal wall serosae, likely resulting from inflammatory responses triggered by leakage of intestinal contents or blood from the wound site. Consistently, adhesion scores on day 14 were significantly higher in the control groups than in the ABP-treated group (**Figure S19, and Table S2**). These outcomes indicate that the ABP effectively mitigates postoperative adhesion formation through its intrinsic anti-adhesive properties.

The ABP patch sustained a stable barrier effect during the critical period of adhesion formation, physically separating tissues prone to adhesion, reducing adhesion formation around normal intestinal tissues, and achieving complete degradation within 14 days 56. This process relies on rapid absorption of interfacial water by the swollen bioadhesive layer, followed by covalent crosslinking between the -NHS active esters and amine groups on the tissue surface. Compared to *in vitro* swelling, the *in vivo* degradation behavior suggests that the degradation of ABP may be attributed not only to the cleavage of the GelMA backbone but also to the complex enzymatic activities in the physiological environment.

To confirm the therapeutic efficacy of the ABP on colon defect healing, we performed histological and immunofluorescence analyses at Day-14 post-repair and compared among the ABP-treated and suture-treated control groups. H&E staining showed that the control group exhibited obvious epithelial injury, scar-like connective tissue formation, abnormal collagen deposition and inflammatory cells infiltration. Additionally, the colon defect in the suture group showed visible traumatic holes caused by local stress concentration and tissue damage caused by suture penetration. In contrast, the ABP-treated group showed a continuous and intact intestinal epithelium, with minimal inflammatory adhesion or collagen deposition (**Figure 7C-E**). These findings demonstrate that the sutureless ABP sealing strategy effectively promoted colon defect healing and prevented additional tissue injury, outperforming conventional suture-based repair.

Inflammatory responses and tissue fibrosis are key contributors to postoperative adhesion formation. Following intestinal wall injury, macrophages are activated and may polarize toward pro-inflammatory phenotypes, while fibrotic remodeling promotes extracellular matrix deposition and adhesion formation. At Day-14, immunofluorescence staining and relative fluorescence intensity analysis showed that the ABP-treated group exhibited significantly reduced expression of collagen I, collagen III, iNOS, and vimentin compared with the control groups (**Figure 7F-I, and Figure S20-21**) These results indicated that ABP-treated group showed reduced fibrosis and inflammatory responses during long-term repair. These results further confirm that the ABP prevents both anastomotic leakage and postoperative adhesion without inducing overt systemic inflammation.

The intrinsic anti-adhesion capability of the ABP could be attributed to the hydrophobicity and biocompatibility of its top layer. The hydrophobic HBP-based top layer forms a stable protective barrier that reduces protein and cell adsorption, thereby preventing attachment of the ABP to surrounding tissues 57. In addition, internal hydrogen bonding within the hydrogel network reduced the availability of surface-active adhesive groups, further decreasing nonspecific adhesion 56. The hydrophobic HBP-based top layer can also self-assemble into an anti-adhesive interface through alkyl chains-driven hydrophobic aggregation, thereby suppressing postoperative adhesion formation by minimizing fibroblast attachment and collagen deposition on the patch surface (**Figure 7J**). Beyond enabling conformal mechanical integration with the target tissue, the ABP also attenuated fibrous capsule formation at the adhesive implant-tissue interface by reducing inflammatory cell infiltration 58. Collectively, these findings suggest that the ABP provides a promising sutureless repair strategy with strong sealing capacity, effective anti-adhesion performance, controlled degradation, and broad translational potential for gastrointestinal defect repair.

## Conclusions

In this study, we developed a novel asymmetric bioadhesive patch (ABP) composed of a hydrophilic and hygroscopic bioadhesive bottom layer for wet-tissue attachment and a non-adhesive polyurethane (PU)/HBP top layer for anti-adhesion protection. The robust adhesion of the ABP to the wet tissue surface is mediated by rapid physical interactions, including interfacial water absorption, hydrogen bonding, electrostatic interactions, and polymer entanglement, followed by covalent crosslinking that further stabilizes the tissue-patch interface over time. Owing to its unique asymmetric design, the ABP simultaneously integrates sutureless sealing, pro-healing activity, anti-adhesion performance, and resistance to tissue deformation, all of which are essential for effective tissue defect repair and functional reconstruction. Overall, this work provides a promising strategy for designing Janus hydrogel bioadhesives that enable non-invasive *in vivo* tissue defect repair while preventing postoperative adhesions. Given its tissue-mimicking biological, mechanical, and biodegradable properties, the ABP may offer a translational platform for gastrointestinal defect repair and other emergency surgical applications.

## Supplementary Material

Supplementary figures and tables, movie legends.

Supplementary video 1.

Supplementary video 2.

Supplementary video 3.

## Data Availability

The data supporting the findings of this study are available from the corresponding author upon reasonable request.

## Abbreviations

AL: Anastomotic leakage
GI: Gastrointestinal
ABP: Asymmetric bioadhesive patch
BP: Bioadhesive patch
Pas: Peritoneal adhesions
HBP: Hyperbranched polyethylene
PAAN: Poly (acrylic acid) grafted with N-hydroxysuccinimide ester
PU: Hydrophobic polyurethane
PAA: Polymer acrylic acid
WCA: Water contact angle
DLP: Digital light processing
Caco-2: Human intestinal epithelial cells
