# Supplementary material for: A robust asymmetric bioadhesive patch for sutureless intestinal repair to prevent anastomotic leakage and postoperative adhesion
Source: Theranostics. 2026 Jun 17;16(13):7641–59. doi: 10.7150/thno.135611 (PMC13295843; doi:10.7150/thno.135611)
Supplement: Supplementary file 1 — Supplementary figures and tables, movie legends. [file thnov16p7641s1.pdf]

A robust asymmetric bioadhesive patch for sutureless intestinal repair to prevent anastomotic leakage and postoperative adhesion

Ruiqi Gao[2†], Fangfang Su[1†], Xiaohua Li[2†], Zhongjie He[1], Yanglin Pan[3], Yaping Zheng[4], Mingzhong Wang[5], Lijun Yuan[1*], Jiahe Liang[1*], and Xiaoqian Li[1]

[1]Department of Ultrasound Medicine, Tangdu Hospital, The Fourth Military Medical University, Xi'an, Shaanxi, 710038, P. R. China

[2]Department of Gastrointestinal Surgery, Xijing Hospital, The Fourth Military Medical University, Xi'an 710032, Shaanxi, China

[3]Xijing Hospital of Digestive Diseases, The Fourth Military Medical University, Xi'an, Shaanxi, 710032, P. R. China

[4]School of Chemistry and Chemical Engineering, Northwestern Polytechnical University, Xi'an, Shaanxi,710129, P. R. China

[5]Department of Pediatrics, Tangdu Hospital, The Fourth Medical University, Xi'an, Shaanxi, 710038, P. R. China

This file includes:

Figure S1 to Figure S21

Table S1 to Table S2

Video S1 to Video S3

[1]Corresponding authors: Xiaoqian Li (xiaoqianli1214@163.com), Lijun Yuan (yuanlj@fmmu.edu.cn), Jiahe Liang (Liangjh2020@stu.xjtu.edu.cn),

[†]These authors contributed equally to this work.

Preparation and characterization

Preparation of PAAN

The PAA solution (10 wt%) was prepared by dissolving PAA (M_w = 240, 000, 25 wt%) in deionized water, followed by stirring at 25 °C. Afterward, 1.2 g EDC and 1.2 g NHS were added into 100 g PAA solution, and then the mixture was stirred for 24 h to facilitate the grafting of NHS functional groups to the PAA chains. Then, the samples were dried by lyophilization. The synthetic route is shown in **Fig. S3.** And, ^{1}H NMR spectra of PAAN is shown in **Fig. S4**.

3D Printing of hydrogel

Precursor solution was utilized to fabricate the designed objects via a digital light processing (DLP) desktop projection-based bioprinter (EFL-BP-8601Pro, Yongqinquan Intelligent Equipment Co., Ltd., Suzhou, China) operating at 385 nm. The printing parameters included a layer thickness of 50-500 mm, a light intensity of 25 $mW·cm^{-2}$, and an exposure time of 1.5 s. SolidWorks software was employed to generate the STL files for these objects. After printing, the obtained constructs were removed, rinsed with ethanol and PBS, and then prepared for subsequent mechanical and tissue adhesion tests.

Mechanical properties tests

Dumbbell-shaped hydrogels (length: 75 mm, width: 5 mm, thickness: 1.0 mm) were subjected to elongation at break measurements according to the GB/T 528-1998 standard.

Histology and immunofluorescence

For histological analysis, the fixed tissues were stored in 70% ethanol and subsequently processed. Staining of the sections was carried out with either hematoxylin and eosin (H&E) or Masson's trichrome.

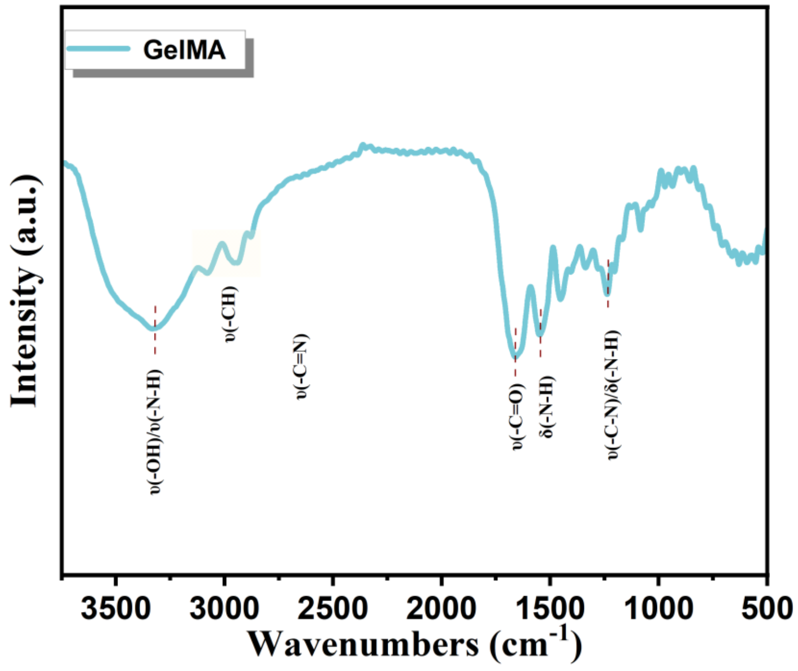

Figure S1 FTIR spectra of GelMA

The typical absorption band around 3338 cm^{-1} is attributed to the ν(O-H) and ν(N-H) stretching vibrations. The peaks in the region 2800-3100 cm^{-1} are ascribed to the stretching vibration of C-H groups. The backbone structure of gelatin is associated with the absorption bands at 1653 cm^{-1} (C=O stretching, amide I), 1544 cm^{-1} (N-H bending coupled to C-H stretching, amide II), and 1239 cm^{-1} (C-N stretching and N-H bending, amide III). The peak at around 1660 cm^{-1} in the spectrum of GelMA, which corresponds to the C=C stretching of the methacrylate groups.

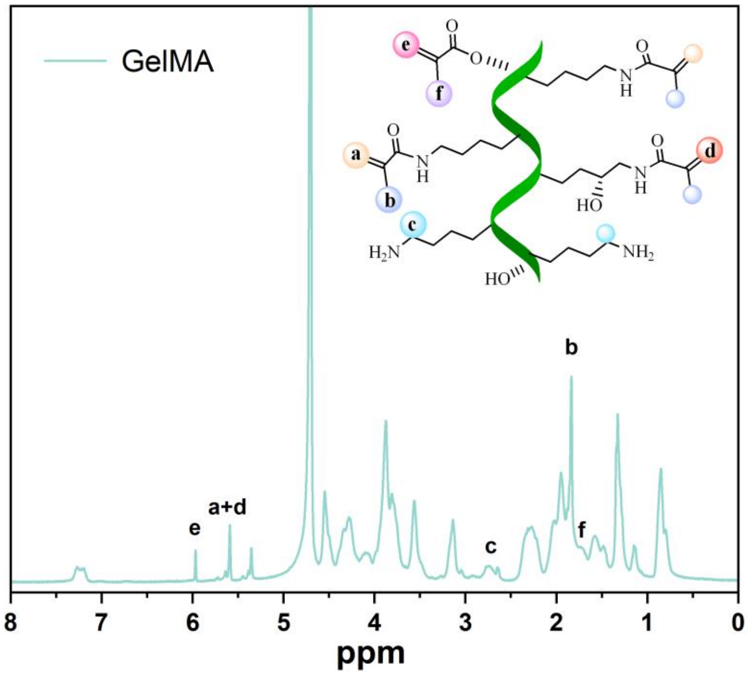

Figure S2 ^{1}HNMR spectra of GelMA. GelMA was prepared by grafting the methacrylate group onto gel through esterification. 5.61 ppm and 5.3 ppm signal peaks in the ^{1}H NMR spectrum of GelMA belonged to the acrylic acid proton of the methacrylic functional group.

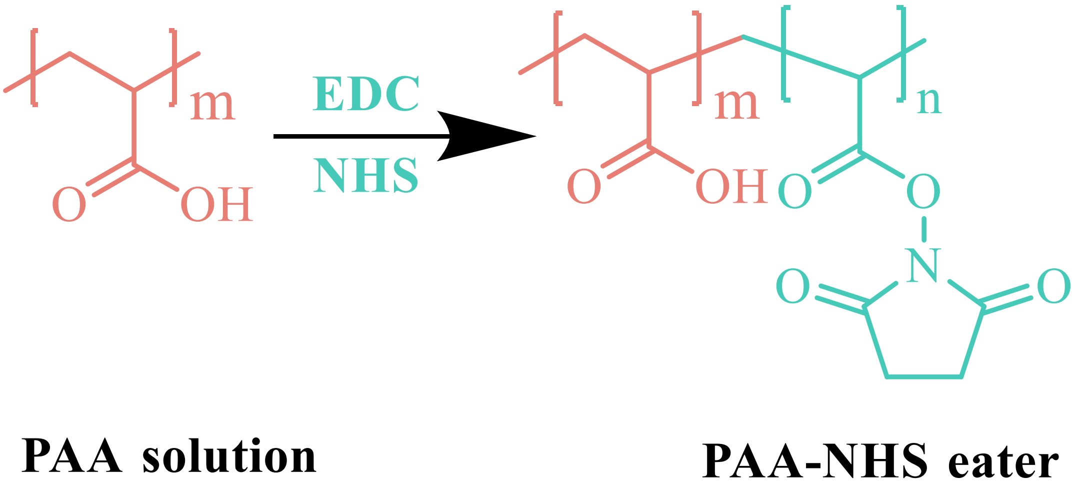

Figure S3 Synthesis of the PAAN solution by grafting N-hydroxysuccinimide (NHS) groups to the PAA chains.

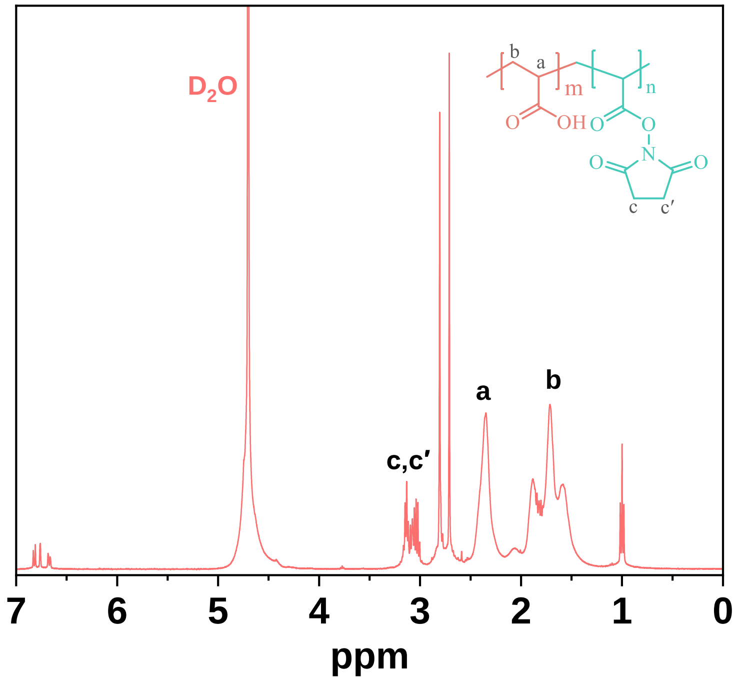

Figure S4 ^{1}HNMR spectra of N-hydroxy succinimide (NHS) groups grafted polyacrylic acid (PAAN).

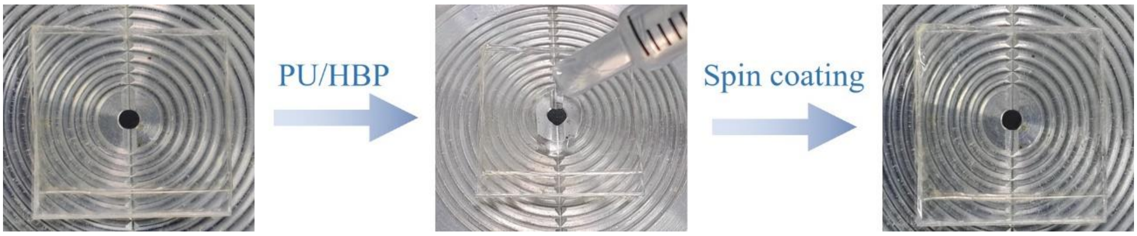

Figure S5 Process diagram of customized Janus for spin coating of the ABP hydrogel.

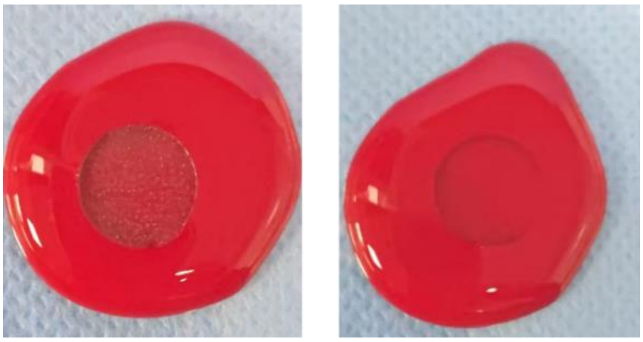

Figure S6 Optical photos of the ABP with and without HBP layer in blood.

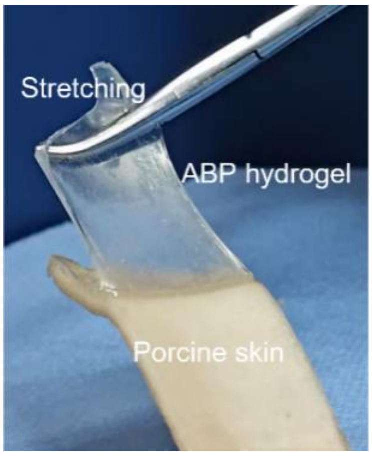

Figure S7 Photographs showing the adhesion behavior of the bottom surface to wet porcine skin surface. The bottom adhesive layer of the ABP tough-bonded to porcine skin.

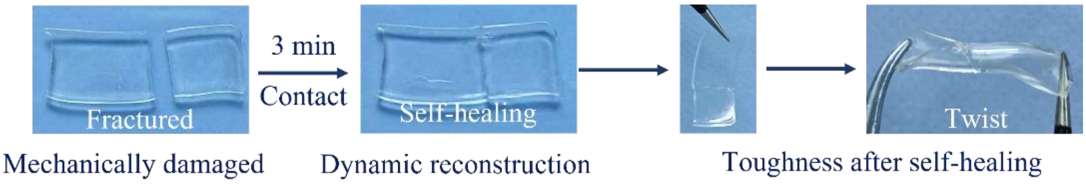

Figure S8 Photographs showing self-healing process of adhesion Janus hydrogel.

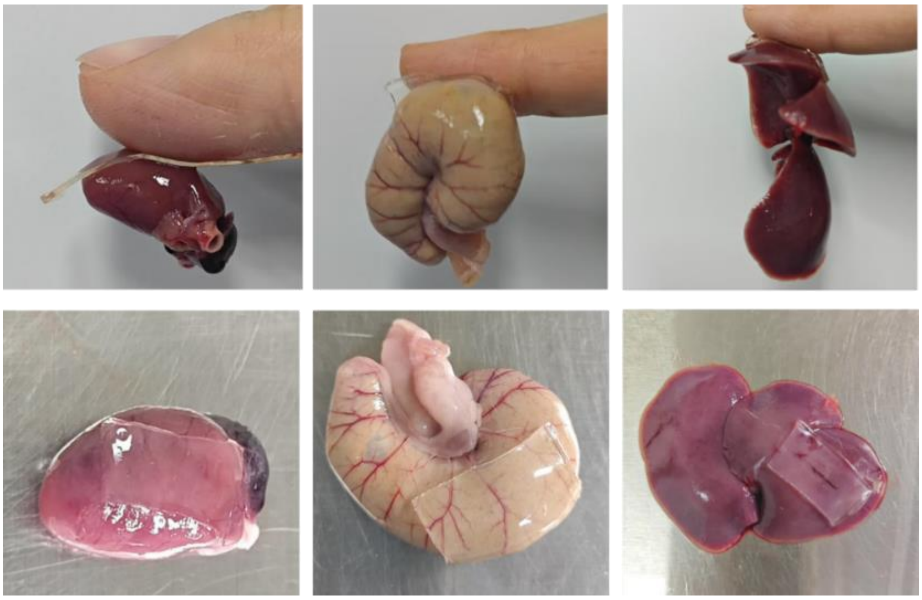

Figure S9 Optical image of ABP to adhesion rat internal organs, including heart, colon, and liver, respectively.

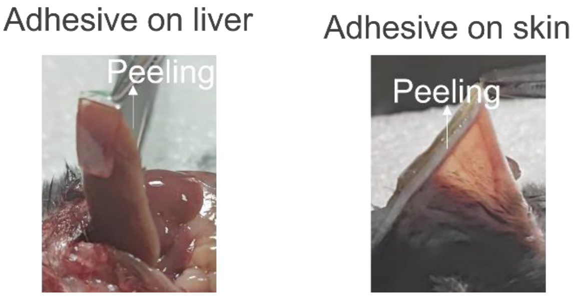

Figure S10 Photographs of the peeling process of the ABP from the rat liver with blood, and skin, respectively. This result demonstrated the ABP tough adhesive to the tissue surface.

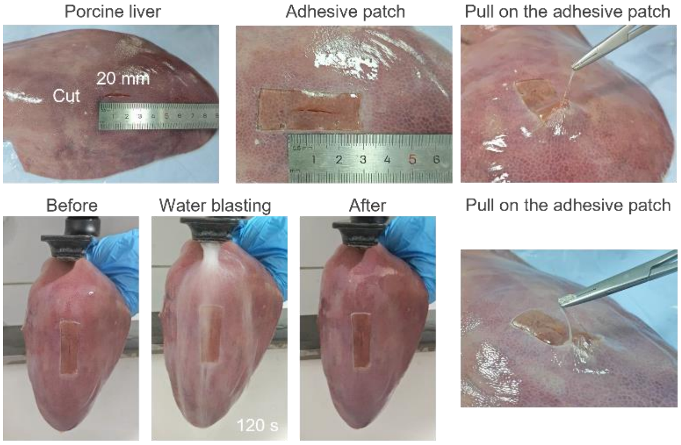

Figure S11 ABP demonstrated robust adhesion to a porcine liver, effectively sealing a 20 mm laceration. Even after being subjected to a large amount of water spraying, the adhesion between ABP and the liver was tough, and constant peeling resulted in the rupture of the porcine liver.

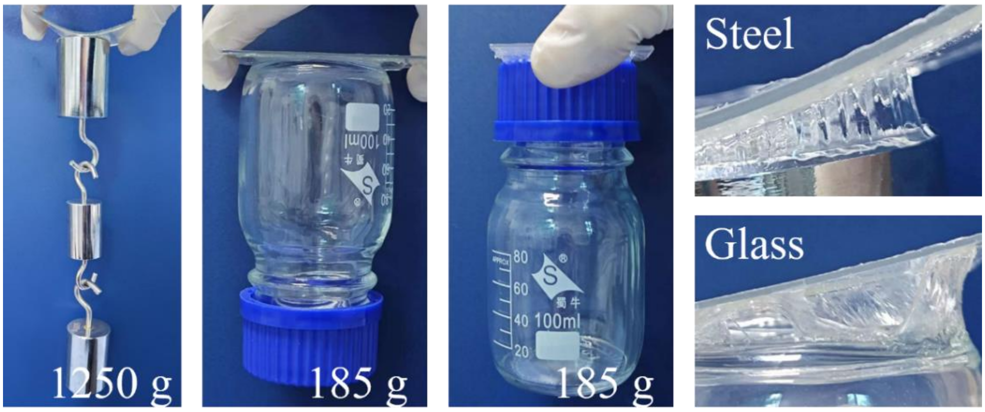

Figure S12 Optical photos of the BP patch lifting a 1250g weights, and a 185 g glass reagent bottle, along with images demonstrating the adhesion and detachment of the BP on the stainless steel and the glass detached surface.

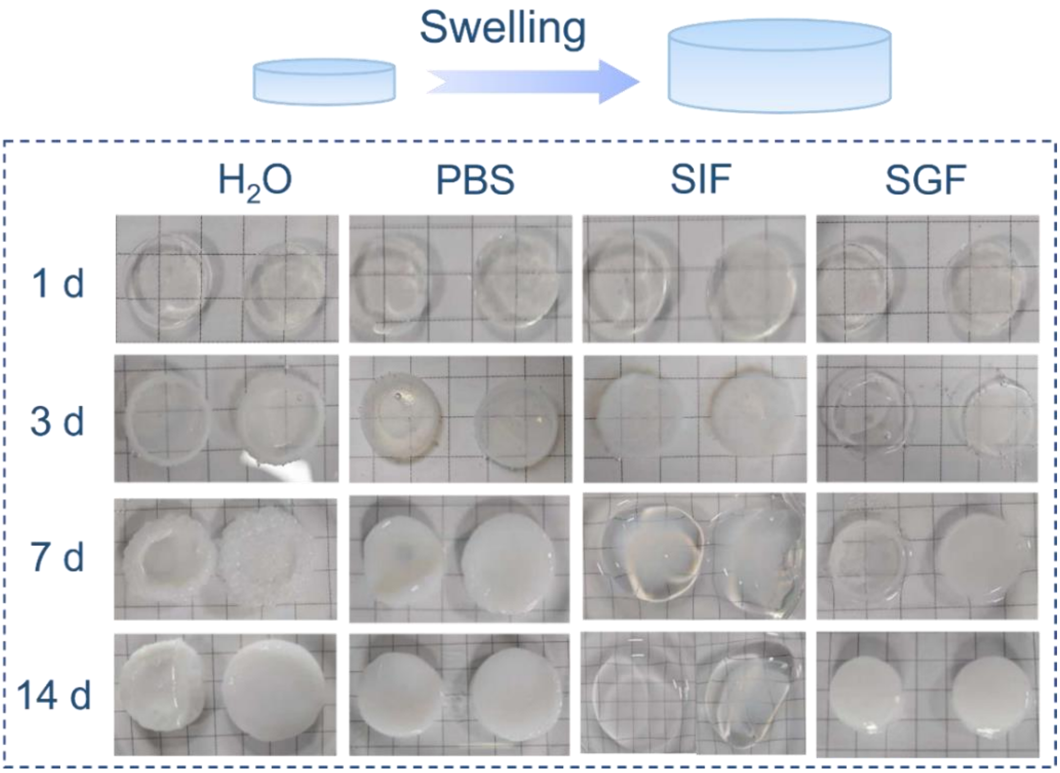

Figure S13 Schematic of the ABP swelling behaviors. Time-dependent swelling behavior of the ABP in H_2O, PBS (pH = 7.4), SGF (pH = 1.2), and SIF (pH = 6.8) at various durations under 37 °C, respectively. Each black square was identical at 10 × 10 mm.

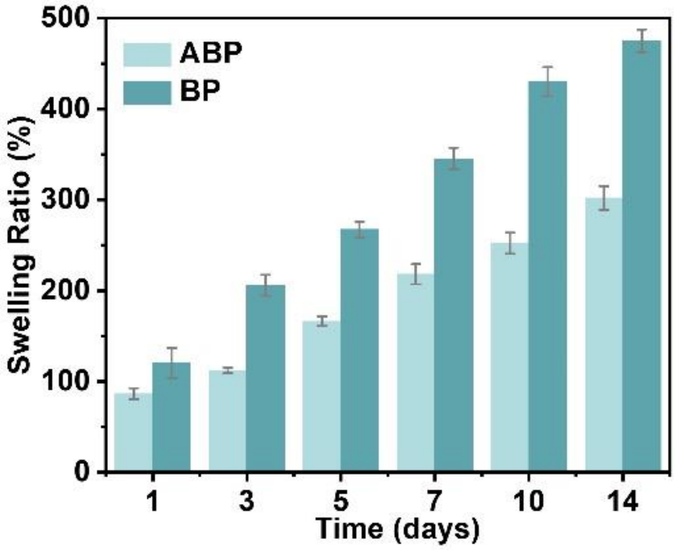

Figure S14 The swelling ratio of the ABP patch and BP patch under SIF (pH = 6.8) at various durations under 37 °C, respectively

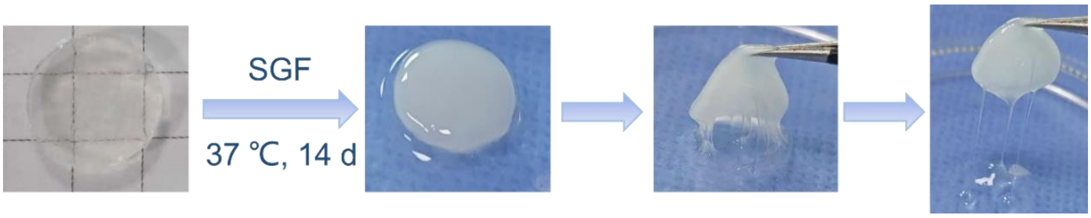

Figure S15 The adhesion behavior of the ABP in SGF after 14 days.

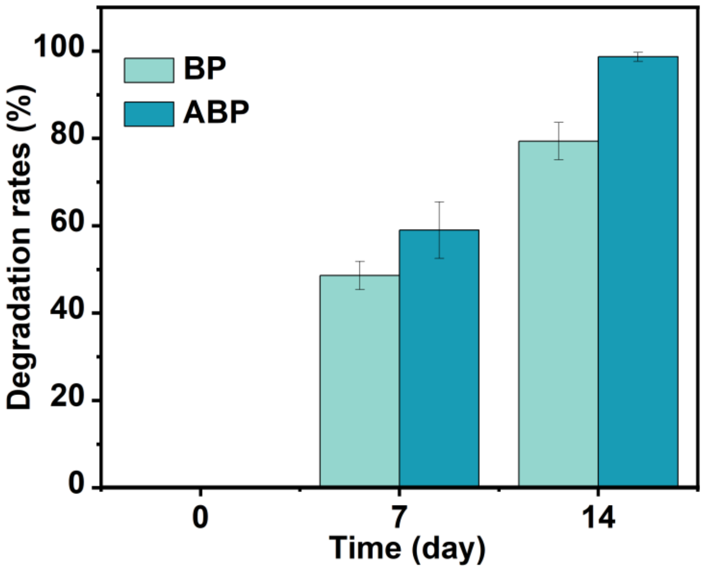

Figure S16. In vitro biodegradation of ABP following subcutaneous implantation in rat abdominal skin (n = 3)

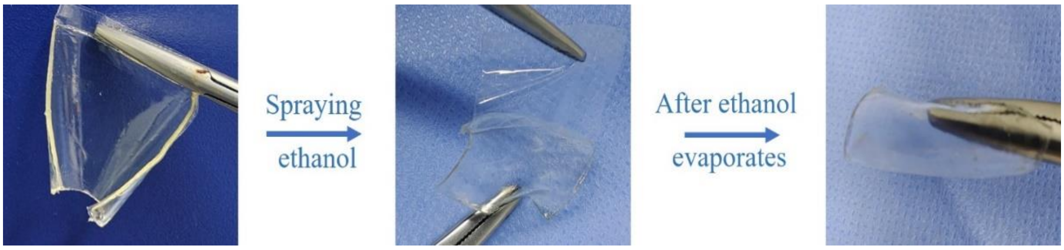

Figure S17. Representative peeling process of the ABP patch from covering, after applying the ethanol.

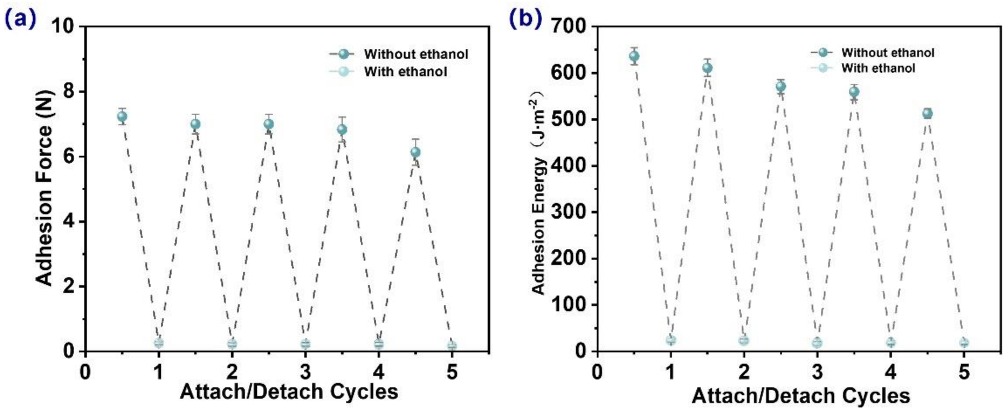

Figure S18. Cyclic ethanol-triggered detachment and adhesion recovery of ABP. We conducted 5 cycles of ethanol detachment and re-adhesion tests by T-peel tests (ASTM F2256). (a) Adhesion force and (b) calculated adhesion energy (interfacial toughness) from 5 attach/detach cycles via T-peel tests (ASTM F2256). The ABP can be easily detached from the skin after ethanol treatment, and the adhesion performance of ABP was restored once the ethanol evaporated.

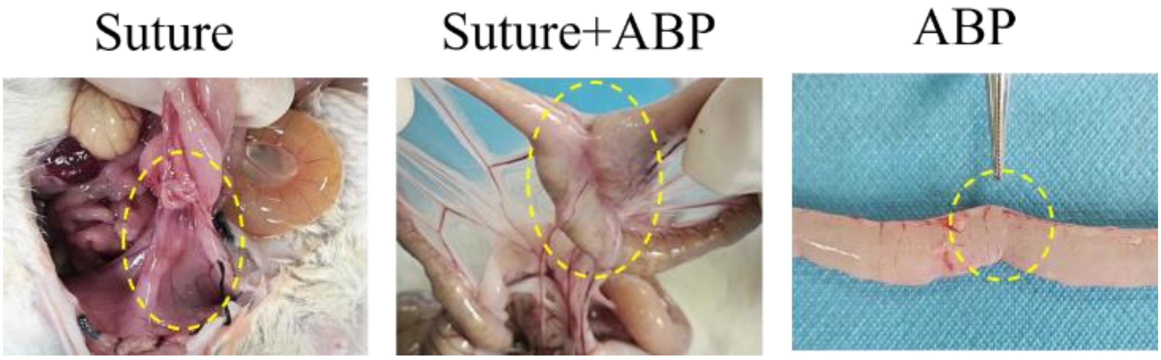

Figure S19 Anti-adhesion efficiency of ABP in a murine cecum-abdominal wall adhesion model. Typical abdominal adhesions in the suture, suture+ABP, and ABP 14 days after operation. Yellow dashed boxes indicate the adhesion sites.

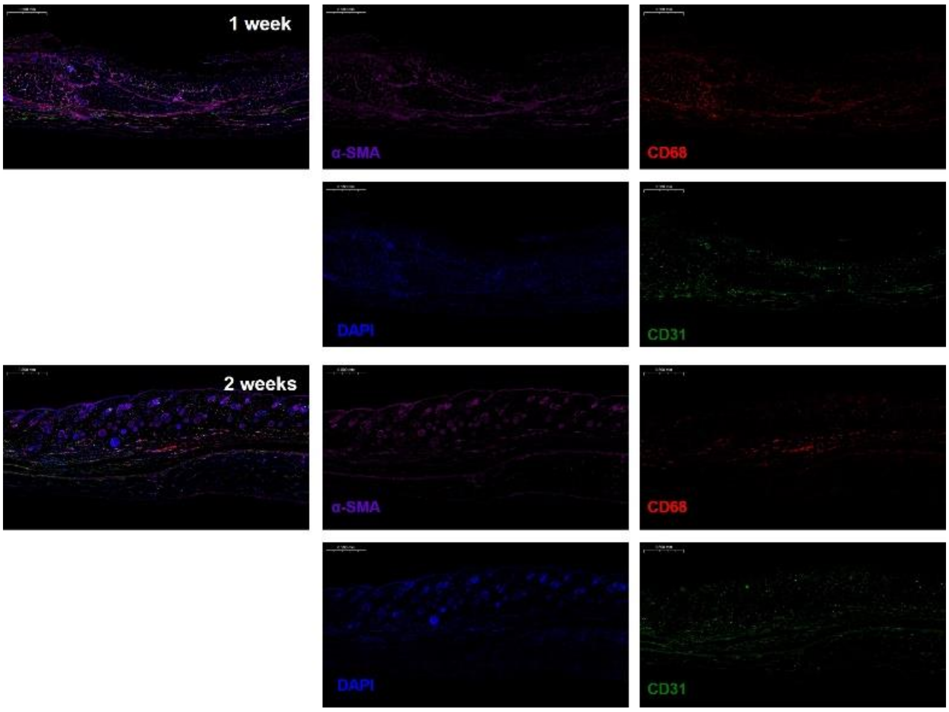

Figure S20 Representative images of CD31, CD68 and α-SMA immunostaining for the subcutaneously implanted ABP patch at 1 week and 2 weeks.

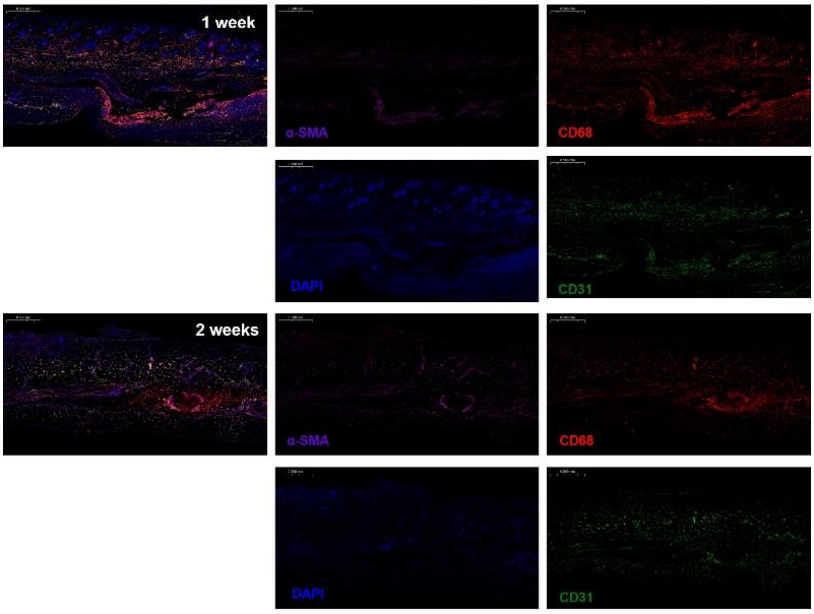

Figure S21 Representative images of CD31, CD68 and α-SMA immunostaining for the subcutaneously implanted BP patch at 1 week and 2 weeks.

Table S1. Mechanical properties of the implants of adhesive patches Comparison of mechanical properties between commercial bioadhesives and reported typical bioadhesives

<table>
<tr><th>Num</th><th colspan="2">Samples names</th><th>Interfacial toughness (J·m^{-2})</th><th>Shear strength (kPa)</th><th>Tensile strength (kPa)</th><th>Burst pressure (kPa)</th><th>Ref</th></tr>
<tr><td>1</td><td colspan="2">GI Patch</td><td>>350 (colon)
>500 (stomach)</td><td>>65 (colon);
>80 (stomach)</td><td>>60 (colon);
>65 (stomach)</td><td>>24 (fully swollen)</td><td>[1]</td></tr>
<tr><td>2</td><td colspan="2">Strain-programmed patch</td><td>350</td><td>115</td><td>-</td><td>-</td><td>[2]</td></tr>
<tr><td>3</td><td colspan="2">Instant tough bioadhesive</td><td>400</td><td>-</td><td>-</td><td>-</td><td>[3]</td></tr>
<tr><td>4</td><td colspan="2">Multifunctional Origami Patch</td><td>536.7±93.4</td><td>56.1±4.7</td><td>65.0±8.0</td><td></td><td>[4]</td></tr>
<tr><td>5</td><td colspan="2">P（AAm-MA-AA）</td><td>124 ± 21</td><td>12</td><td></td><td>>18.6</td><td>[5]</td></tr>
<tr><td>6</td><td colspan="2">AG-PEG</td><td>≈260 (skin)
≈120 (intestines)
≈100 (stomach)
≈200 (muscle)
≈190 (heart)
≈80 (liver)</td><td>≈70(skin)
≈50(intestines)
≈50(stomach)
≈60(muscle)
≈60 (heart)
≈25 (liver)</td><td>-</td><td>≈50</td><td>[6]</td></tr>
<tr><td>7</td><td colspan="2">OD/GM@PG</td><td>325</td><td>98</td><td>-</td><td>12.7</td><td>[7]</td></tr>
<tr><td>8</td><td colspan="2">Instant tough bioadhesive</td><td>> 400 J·m^{-2}
（5s） wet dynamic tissues</td><td>-</td><td>-</td><td>-</td><td>[3]</td></tr>
<tr><td>9</td><td colspan="2">DST</td><td>> 750 (skins)
> 580 (small intestine)
> 450(stomach)
> 570 (muscle)
> 340 (heart)
> 190 (liver)</td><td>> 120 (skins)
> 80 (small intestine)
> 70 (stomach)
> 80 (muscle)
> 70 (heart)
> 20 (liver)</td><td>> 160 (wet porcine skins)</td><td>-</td><td>[8]</td></tr>
<tr><td>10</td><td colspan="2">UIHA</td><td>44.6±14.4 (porcine skin)
115.2±13.8 (porcine skin)</td><td>-</td><td>-</td><td>16±1 (ambient condition)
18.1±0.5 (underwater)</td><td>[9]</td></tr>
<tr><td>11</td><td colspan="2">e-bioadhesive interface</td><td>>420 (heart);
>270 (skin);
>230 (muscle);
>260 (sciatic nerve)</td><td>>110 (heart);
>60 (skin);
>50 (muscle);
>70 (sciatic nerve)</td><td>-</td><td>-</td><td>[10]</td></tr>
<tr><td>12</td><td colspan="2">MAH-600</td><td>>300</td><td>>75 (porcine skin)</td><td>-</td><td>25.9</td><td>[11]</td></tr>
<tr><td>13</td><td colspan="2">ATGels</td><td>≈300</td><td>40-60</td><td>-</td><td>33.9</td><td>[12]</td></tr>
<tr><td>14</td><td colspan="2">HA-PEG gel （7%）</td><td>-</td><td>-</td><td>27.6 ± 3.9 kPa</td><td>22.7± 4</td><td>[13]</td></tr>
<tr><td>15</td><td colspan="2">SF patch</td><td>-</td><td>-</td><td>50.43 ± 4.30</td><td>6.7±1</td><td>[14]</td></tr>
<tr><td rowspan="5">16</td><td rowspan="4">Commercially bioadhesives</td><td>Histoacryl</td><td>≈31.4 (colon)</td><td>≈11.8 (colon)</td><td>≈11.9 (colon)</td><td>≈12</td><td rowspan="5">[1, 13]</td></tr>
<tr><td>Coseal</td><td>≈11.3 (colon)</td><td>≈3.8 (colon)</td><td>≈6.4 (colon)</td><td>≈3.2</td></tr>
<tr><td>Tisseel</td><td>≈26.8 (colon)</td><td>≈9.1 (colon)</td><td>≈10.6 (colon)</td><td>≈5.0</td></tr>
<tr><td>Fibrin glue</td><td>< 22 (wet tissues)</td><td>< 12 (wet tissues)</td><td></td><td>9.8±2</td></tr>
<tr><td colspan="2">Suture</td><td>-</td><td>-</td><td>-</td><td>≈78</td></tr>
<tr><td rowspan="2">17</td><td rowspan="2">Human</td><td>Blood pressure</td><td></td><td></td><td></td><td>10.6-15.9</td><td rowspan="2">[12]</td></tr>
<tr><td>intra-gastrointestinal pressure</td><td></td><td></td><td></td><td>0.67-3.3</td></tr>
<tr><td>18</td><td colspan="2">ABP</td><td>>685.7 (colon)
>539.5 (stomach)
>748.8 (skin)</td><td>>82.5 (colon)
>85.9 (stomach)
>89.7 (skin)</td><td></td><td>56.1</td><td>This work</td></tr>
</table>

Table S2. Clinical adhesion scoring criteria

Criteria[a]	Adhesion extent[b]	Adhesion type	Adhesion tenacity[c]	Group
0	No adhesion	No adhesion	No adhesion	ABP
1	0-25% coverage	Dense	Easily fall apart	
2	25-50% coverage	Thick	Require traction	
3	50-75% coverage	Capillaries present	Require sharp dissection	suture<br>suture+ABP
4	75-100% coverage	/		

[a] Clinical adhesion score consists of adhesion extent, adhesion type and adhesion tenacity

[b] The ratio of adhesion area to the total area of implant surface.

[c] Force or surgical tools are required to detach the adhered tissues.

Supplementary Video:

Video S1. Strong adhesion of the ABP patch demonstrated by a 3 kg weight-bearing test between two pieces of porcine skin" to further demonstrate the adhesion property of the ABP patch;

Video S2. Representative peeling process of the ABP patch from human finger skin, after applying the ethanol;

Video S3. Representative peeling process of the ABP patch from human arm skin, after applying the ethanol.

References

1. Wu J, Yuk H, Sarrafian TL, Guo CF, Griffiths LG, Nabzdyk CS, et al. An off-the-shelf bioadhesive patch for sutureless repair of gastrointestinal defects. Sci Transl Med. 2022; 14: 2857-69.

2. Theocharidis G, Yuk H, Roh H, Wang L, Mezghani I, Wu J, et al. A strain-programmed patch for the healing of diabetic wounds. Nat Biomed Eng. 2022; 6: 1118-33.

3. Chen X, Yuk H, Wu J, Nabzdyk CS, Zhao X. Instant tough bioadhesive with

triggerable benign detachment. Proc Natl Acad Sci USA. 2020; 117: 15497-503.
4. Wu SJ, Yuk H, Wu J, Nabzdyk CS, Zhao X. A multifunctional origami patch for minimally invasive tissue sealing. Adv Mater. 2021; 33: 2007667.
5. Anthis AHC, Hu X, Matter MT, Neuer AL, Wei K, Schlegel AA, et al. Chemically stable, strongly adhesive sealant patch for intestinal anastomotic leakage prevention. Adv Funct Mater. 2021; 31: 2007099.
6. Wang H, Cheng J, Sun F, Dou X, Liu J, Wang Y, et al. A super tough, rapidly biodegradable,ultrafast hemostatic bioglue. Adv Mater. 2023; 35: 2208622.
7. Lv Y, Cai F, Zhao X, Zhu X, Wei F, Zheng Y, et al. Bioinspired microstructured janus bioadhesive for the prevention of abdominal and intrauterine adhesions. Adv Funct Mater. 2024; 13: 2402268.
8. Yuk H, Varela CE, Nabzdyk CS, Mao X, Padera RF, Roche ET, et al. Dry double-sided tape for adhesion of wet tissues and devices. Nat. 2019; 575: 169-74.
9. Liu Y, Guan G, Li Y, Tan J, Cheng P, Yang M, et al. Gelation of highly entangled hydrophobic macromolecular fluid for ultrastrong underwater in situ fast tissue adhesion. Sci Adv. 2022; 8: 9744-9757.
10. Deng J, Yuk H, Wu J, Varela CE, Chen X, Roche ET, et al. Electrical bioadhesive interface for bioelectronics. Nat Mater. 2020; 20: 229-36.
11. Wang H, Yi X, Liu T, Liu J, Wu Q, Ding Y, et al. An integrally formed janus hydrogel for robust wet-tissue adhesive and anti-postoperative adhesion. Adv Mater. 2023; 35: 2300394.
12. Chen X, Zhang J, Chen G, Xue Y, Zhang J, Liang X, et al. Hydrogel bioadhesives with extreme acid-tolerance for gastric perforation repairing. Adv Funct Mater. 2022; 32: 2202285.
13. Ren H, Zhang Z, Cheng XL, Zou Z, Chen XS , He CL. Injectable, self-healing hydrogel adhesives with firm tissue adhesion and on-demand biodegradation for sutureless wound closure. Sci Adv. 2023; 9: 4327.
14. Zheng M, Du LZ, Zheng J, Tao J, Wu Y, Qiu L, et al. Highly tough and conformal silk-based adhesive patches for sutureless repair of gastrointestinal and peripheral nerve defects. Bioact Mater. 2025; 53: 1-19.
